# Dynamics of Gut Microbiota and Clinical Variables after Ketogenic and Mediterranean Diets in Drug-Naïve Patients with Type 2 Diabetes Mellitus and Obesity

**DOI:** 10.3390/metabo12111092

**Published:** 2022-11-10

**Authors:** Andrea Deledda, Vanessa Palmas, Vitor Heidrich, Michele Fosci, Mauro Lombardo, Giulia Cambarau, Alessio Lai, Marietta Melis, Elisabetta Loi, Andrea Loviselli, Aldo Manzin, Fernanda Velluzzi

**Affiliations:** 1 Obesity Unit, Department of Medical Sciences and Public Health, University of Cagliari, 09124 Cagliari, Italy; 2 Department of Biomedical Sciences, University of Cagliari, 09042 Monserrato, Italy; 3 Departamento de Bioquímica, Instituto de Química, Universidade de São Paulo, São Paulo 05508-900, Brazil; 4 Centro de Oncologia Molecular, Hospital Sírio-Libanês, São Paulo 01308-050, Brazil; 5 Endocrinology Unit, Department of Medical Sciences and Public Health, University of Cagliari, 09042 Monserrato, Italy; 6 Department of Human Sciences and Promotion of the Quality of Life, San Raffaele Roma Open University, 00166 Rome, Italy; 7 Diabetologia, P.O. Binaghi, ASSL Cagliari, 09126 Cagliari, Italy

**Keywords:** ketogenic diet, Mediterranean diet, type 2 diabetes mellitus, obesity, body composition, glycometabolic status, physical activity, quality of life, gut microbiota, 16S rRNA

## Abstract

Type 2 diabetes mellitus (T2DM), the most common form of diabetes, is a progressive chronic metabolic disease that has increasingly spread worldwide, enhancing the mortality rate, particularly from cardiovascular diseases (CVD). Lifestyle improvement through diet and physical activity is, together with drug treatment, the cornerstone of T2DM management. The Mediterranean diet (MD), which favors a prevalence of unprocessed vegetable foods and a reduction in red meats and industrial foods, without excluding any food category, is usually recommended. Recently, scientific societies have promoted a very low-calorie ketogenic diet (VLCKD), a multiphasic protocol that limits carbohydrates and then gradually re-introduces them, with a favorable outcome on body weight and metabolic parameters. Indeed, gut microbiota (GM) modifications have been linked to overweight/obesity and metabolic alterations typical of T2DM. Diet is known to affect GM largely, but only a few studies have investigated the effects of VLCKD on GM, especially in T2DM. In this study, we have compared anthropometric, biochemical, lifestyle parameters, the quality of life, and the GM of eleven patients with recently diagnosed T2DM and overweight or obesity, randomly assigned to two groups of six and five patients who followed the VLCKD (KETO) or hypocaloric MD (MEDI) respectively; parameters were recorded at baseline (T0) and after two (T2) and three months (T3). The results showed that VLCKD had more significant beneficial effects than MD on anthropometric parameters, while biochemical improvements did not statistically differ. As for the GM, despite the lack of significant results regarding the alpha and beta diversity, and the Firmicutes/Bacteroidota ratio between the two groups, in the KETO group, a significant increase in beneficial microbial taxa such as Verrucomicrobiota phylum with its members Verrucomicrobiae, Verrucomicrobiales, Akkermansiaceae, and *Akkermansia*, Christensenellaceae family, *Eubacterium* spp., and a reduction in microbial taxa previously associated with obesity (Firmicutes and Actinobacteriota) or other diseases (*Alistipes*) was observed both at T2 and T3. With regards to the MEDI group, variations were limited to a significant increase in Actinobacteroidota phylum at T2 and T3 and Firmicutes phylum at T3. Moreover, a metagenomic alteration linked to some metabolic pathways was found exclusively in the KETO group. In conclusion, both dietary approaches allowed patients to improve their state of health, but VLCKD has shown better results on body composition as well as on GM profile.

## 1. Introduction

Type 2 diabetes mellitus (T2DM), the most common form of diabetes, is a progressive chronic metabolic disease with a growing prevalence worldwide, which leads to increased mortality, in particular from cardiovascular diseases (CVD) [1].

T2DM, whose main feature is hyperglycemia [2,3], is caused by a combination of defects in the secretion and peripheral action of insulin. The loss of glycemic homeostasis is linked to several genetic and environmental causes; in particular, genetic [4] and epigenetic susceptibility [5] predispose to the development of T2DM, while the environmental factors are fundamental to the onset and clinical manifestations of the disease. Among the environmental factors, an incorrect and unhealthy lifestyle (sedentary behavior, high-caloric, and poor eating habits), the consequent condition of overweight or obesity [6], and especially the related deficit of metabolically active lean mass play a key role.

Other factors, such as sleep quality, mental disturbances, or psychiatric disorders, frequently observed in obesity [7,8], are associated with increased diabetes risk [9].

Regarding obesity, the risk of T2DM is more proportional to the presence of visceral and ectopic fat than to weight excess, usually assessed by Body Mass Index (BMI) value. Indeed, visceral and ectopic fat deposition is associated with adipocyte dysfunction, adipokine dysregulation, inflammation, and insulin resistance [10,11].

Insulin resistance (IR), defined as a reduction of the biological efficiency of insulin and insulin sensitivity in peripheral tissues, is the basis of the pathophysiology of T2DM [12]. In addition, the alteration of the leptin signal, caused by an increased release of leptin from excess adipose tissue, can promote the loss of the hypothalamic control over energy intake and expenditure and carbohydrates metabolism [13,14,15], with consequent alterations in endocrine-metabolic regulation and in nutrients partitioning [16].

Among the causes of the altered metabolic control typical of T2DM, the most known is the release of proinflammatory cytokines and free fatty acids (FFA) by adipose tissue infiltrated by macrophages [17] and a proinflammatory diet rich in packaged foods [18], industrial fructose [19], low-quality fats [20], and Advanced Glycation End Products (AGEs) [21]. Other emerging causes are dysfunctional mitochondria [22], which alter pancreatic insulin secretion [23], oxidative stress, which amplifies inflammation [24], exposure to endocrine disruptors, especially during intrauterine life [25,26], and misalignment of circadian rhythms [27].

The gut microbiota (GM), the bacterial community resident in the intestine, which has a well-known effect and link with the state of health, and whose alterations are associated with several health problems, such as metabolic, allergic, and autoimmune diseases [28,29], has recently acquired a primary role in the pathogenesis of IR and T2DM. In particular, an altered microbiota pattern can release proinflammatory factors that, in the presence of an altered intestinal permeability, enter the bloodstream favoring a constant immune and inflammatory response. In addition, it can affect glucose metabolism by regulating insulin sensitivity, glycogen storage, glucose uptake, and gluconeogenesis enzymes; fatty acid oxidation, synthesis, and energy expenditure; pharmacodynamics and pharmacokinetics of diabetes drugs [30].

Moreover, there is a complex bidirectional relationship between body weight and GM [31], and despite the not univocal data [32], the main characteristics of overweight or obesity are an increased *Firmicutes*/*Bacteroidetes* ratio and a reduced diversity [33].

Regarding T2DM, *Bifidobacterium*, *Bacteroides*, *Faecalibacterium*, *Akkermansia*, and *Roseburia* were observed to be negatively associated, whereas *Ruminococcus*, *Fusobacterium*, and *Blautia* are positively associated with the disease [30].

All the aforementioned factors interact with each other by reducing insulin sensitivity and favoring the condition of hyperglycemia, which causes inflammation *per se*, progression of the disease, and damage to the vascular endothelium, leading to microvascular or macrovascular complications and increased CV risk [6,34,35,36]. In addition, diabetic patients are also more susceptible to tumors [37] and neurodegenerative diseases [38].

Because of its complexity, the treatment of T2DM requires a multidimensional patient-centered approach [39]. Current international guidelines recommend lifestyle improvements by means of dietary interventions and physical activity training, pharmacological therapies based on different classes of glucose-reducing drugs [40], nutraceutical supplements [41], and bariatric surgery [42] as therapeutic strategies [2].

### 1.1. Nutritional Treatment in T2DM

In recent years, several scientific associations have developed various guidelines concerning the nutritional treatment of T2DM. In contrast with previous guidelines, which mainly focused on caloric intake without particular indications of foods to be preferred, the common feature of the most recent guidelines is an improvement in the quality of diets and the sources of macronutrients [43,44,45].

Several dietary approaches have been shown to improve the metabolic status [46,47]; however, the Mediterranean diet (MD), predominantly based on plant and unprocessed foods, rich in healthy monounsaturated fats (MUFA) derived from extra-virgin olive oil, and low in saturated fats derived from red meats, is generally considered the most appropriate and the best dietary model to reduce CV risk [48,49].

Among the beneficial biological effects attributed to MD, anti-inflammatory, antihypertensive, antidiabetic, and antiatherogenic effects are demonstrated by both mechanistic and intervention trials. The MD also appears to reduce the incidence of numerous types of cancers [50,51,52] and is associated with the reduction of the risk of dermatological [53,54,55], allergic, and asthmatic [56] diseases. Furthermore, MD also has a positive impact on pollution and environmental sustainability, and these features also contribute to reducing the risk of CVD and promoting overall wellness [57,58].

Finally, it has been shown that MD can positively modulate GM composition and diversity [59], particularly in overweight and obese patients [60].

Regarding T2DM, MD, due to its antioxidant and anti-inflammatory properties, as well as the positive changes in GM, has proven to be beneficial in both the prevention and the progression of the disease [61].

Recently, a new agreement regarding the definition of remission from T2DM has been reached [62]. Both aggressive dietary treatment [63,64] and bariatric surgery [42] have shown to induce remission of T2DM, and in general, a weight loss of more than 10% could lead to remission in newly diagnosed T2DM patients [65].

It is not clear which diet can increase the chances of remission, but the reduction and/or exclusion of junk foods and the consumption of fresh and unprocessed foods appear to play an important role [62]. Furthermore, a recent meta-analysis reports that dietary programs which include a hypocaloric formula ‘total diet replacement’ were the most effective in inducing T2DM remission in one year [66].

### 1.2. Ketogenic Diet

The Ketogenic diet (KD) is defined as a diet capable of inducing a state of ketosis, namely the presence of physiologically relevant blood ketones levels (~4 mmol/L), due to the action of the liver, which condenses the overproduced acetyl-CoA to form ketone bodies (KBs) [67].

Ketosis can be induced by a few days of fasting or a drastic reduction of carbohydrate intake (<50 g/day). In these conditions, KBs begin to be used as an alternative energy source by the Central Nervous System (CNS) [67].

The therapeutic role of KD was reported nearly a century ago, when it was initially used as a protocol to manage severe epilepsy [68], and in recent years, growing evidence has indicated KD as an adjuvant treatment for neurodegenerative and psychiatric diseases [69].

Since the 1960s, KD has been used to encourage weight loss, becoming more popular in the 70s and 80s as the slimming protocol Protein-Sparing Modified Fasting (PSMF), introduced by Blackburn. To achieve quick and significant weight loss, KD is used in its very low-calorie intake form (VLCKD) [70].

The large caloric deficit favors the oxidation of storage fats as the main source of energy for the body [71], and at the same time, ketosis suppresses appetite [72] while promoting the preservation of lean mass; nevertheless, the effect of VLCKD on lean mass does not seem different from that obtained with other weight loss interventions [73].

Although some perplexities persist in the scientific community, in recent years, several scientific societies have suggested the use of VLCKD as a safe and effective way to manage weight loss in people affected by obesity with and without T2DM, specifying that this diet can lead to the improvement of metabolic parameters in a similar and sometimes better way than the MD [67,74], and releasing specific guidelines with detailed indications and contraindications [71,73,75,76,77,78].

Regarding the effects of KD on the GM, they have been predominantly reported in animal models or, in humans, in case of neurological diseases, in which microbiota modulation appears to be one of the mechanisms contributing to the neurological effects of KD, justifying its use in treating epilepsy, migraines and, potentially, other neurological and psychiatric diseases [79,80,81].

On the contrary, there are still few studies that have investigated GM and KD for weight loss, especially in T2DM patients [82,83].

Due to the paucity of data on this issue, the aim of our study is to evaluate the short-term effects on GM of a lifestyle intervention focused on nutritional treatment in a sample of newly diagnosed T2DM patients associated with obesity; the study focuses in particular on the comparison of the effects of two dietary models, MD and VLCKD, also evaluating the effectiveness of the two diets on anthropometric, metabolic, lifestyle, and quality of life parameters.

## 2. Materials and Methods

### 2.1. Study Design and Characteristics of the Sample

The study included twelve patients (6 Males and 6 Females) with an age range between 45 and 65, recruited among the outpatients attending the Obesity Unit of the AOU of Cagliari (Italy) and the Diabetology Unit of the PO Binaghi (ASSL of Cagliari, Italy) after a new diagnosis of T2DM, from March to May 2020. The COVID-19 pandemic, and especially the lockdown period, limited the recruitment of patients; thus, the total number was slightly lower than that provided in the original design of this study. The data were collected at baseline (T0) and after two (T2) and three months (T3).

The study was approved by the Local Ethical Committee of the AOU of Cagliari (Prot. N. PG/2020/9435) and carried out according to the Code of Ethics of the World Medical Association (Declaration of Helsinki) for experiments involving humans. The selected patients were clearly and comprehensively informed about the purpose and the design of the study, and they signed an informed consent to join the research.

The Inclusion criteria were the presence of a newly diagnosed and not complicated T2DM (according to IDF/ADA criteria) [2,3], a glycosylated hemoglobin (HbA1c) value of 6.5–8.9%, a Body Mass Index (BMI) ≥28 Kg/m^2^, and the condition of drug-naïve patients for T2DM.

The exclusion criteria were the presence of T1DM, serious heart diseases, severe and/or uncontrolled hypertension, severe or uncompensated kidney, liver, or thyroid diseases, painful pathologies with severe functional limitations, tumors in chemo/radiotherapy, severe psychopathology, gastrointestinal diseases, therapy with corticosteroids, proton pump inhibitors, antimicrobials, prebiotic or probiotic intake, and any dietary supplements or participation to other dietary regimes in the three months preceding the sample collection.

After the enrollment, at baseline (T0), the selected patients underwent a multidimensional evaluation including clinical, metabolic, anthropometric, lifestyle, and quality of life assessment. At the same time, the analysis of GM was performed.

Patients were then randomly assigned to the specific nutritional intervention (NI): 6 patients (3M, 3F) were assigned to a low-calorie MD according to ADA guidelines (MEDI group) [46] and 6 patients (3M, 3F) to a VLCKD diet (KETO group) [76].

During the first month of NI, one female patient of the MEDI group was excluded because of COVID-19 infection; thus, the sample study included eleven patients (6M, 5F): 6 (3M, 3F) in the KETO group and 5 (3M, 3F) in the MEDI group.

All patients underwent the same scientific procedural rigor, and the same conditions (operators, environment, breaks/holidays, etc.) were guaranteed in the two groups to limit the influence of independent variables and to consolidate the reliability of the results.

The same evaluation performed at T0 was carried out three months after the baseline assessment (T3) for the comparison of results. After the first and second month of NI (T1, T2), a short anthropometric and clinical (glycometabolic status) evaluation and a brief interview aimed at ascertaining the adherence to the nutritional program was performed to analyze any critical issues or strengthen motivation, particularly, in KETO patients the T1 and T2 aimed at monitoring the state of ketosis, and modulating their nutritional treatment structured on well-defined phases, as described in the paragraph “Dietary protocols.” Moreover, at T2 (end of the ketosis phases in the KETO group), the GM analysis was repeated in both study groups.

### 2.2. Clinical, Metabolic, Anthropometric, Lifestyle, and Health Status Evaluation

The clinical examination, performed by the endocrinologist, included the collection of a full medical history, detailed information on drug use, physical examination, standardized Blood Pressure (BP) and Heart Rate (HR) measurement, as well as the quality of life assessment.

The metabolic assessment consisted of a 12 h fasting blood sample for determination with standard methods of fasting plasma glycemia (FPG), glycosylated hemoglobin (HbA1c), C peptide, total cholesterol, HDL (LDL calculated by Friedwald’s formula), total triglycerides, creatininemia, aspartate aminotransferase (AST), alanine aminotransferase (ALT), uricemia, blood count, protein electrophoresis, sodium (Na^+^), potassium (K^+^), calcium (Ca^2+^), thyroid stimulating hormone (TSH), urine test, microalbuminuria determinations.

The anthropometric measurements were taken by the same expert nutritionist according to current standards [84]. Weight expressed in kilograms (kg) was measured with an impedance scale (Model TANITA BC420 MA, Amsterdam, The Netherlands) while patients were in a fasting state, wearing only light clothes. Height, expressed in centimeters (cm), was measured by a stadiometer (SECA, Hamburg, Germany) on barefoot patients with heels together and the body kept in an upright position. The reading was approximated to the nearest cm.

The Body Mass Index (BMI) was calculated according to the weight (kg) to height squared ratio (kg/m^2^). The waist circumference (WC), expressed in cm, was measured according to the NHANES III protocol [84].

The analysis of body composition (BIA) at T0 and T3 was carried out by the bioelectrical impedance analyzer (Mascaretti, Ancona, Italy). The exam was performed as suggested by the European Society of Parenteral and Enteral Nutrition (ESPEN) [85]. The data obtained corresponds with the resistance and reactance, which inserted in the software indicated fat mass (FM%), fat-free mass (FFM kg), and phase angle (phA°).

The intermediate anthropometric evaluation (T1, T2) consisted exclusively of the weight, BMI, and WC measurements.

The lifestyle evaluation, carried out by the nutritionist during an individual interview, included the nutritional and physical activity level assessment.

The nutritional assessment consisted of the administration of a standardized and validated questionnaire aimed at establishing the degree of adherence to the MD through a score (Mediterranean Diet Score, MDS, range 0–55) [86] and a detailed report in the form of a food diary in the three days of the weekend preceding the visit, analyzed using the Winfood^®^ software (Medimatica, Colonnella, Italy). More specifically, the description of the Mediterranean Diet Score, and food diary analysis, can be found as Supplementary Materials in the section “Nutritional evaluation”.

The administration of the International Physical Activity Questionnaire (IPAQ), used in its short version, allowed the ascertainment of the level of physical activity (PAL) and the degree of sedentariness. The PAL was calculated according to the estimated energy expenditure derived from the reported physical activity (vigorous, moderate, and walking) and expressed in Metabolic Equivalent of Task-minutes per week (METs/w), while the sedentary behavior was estimated by assessing the average daily sitting time (h/day) [87].

The assessment of the Quality of Life and perceived health status was performed through the administration of the standardized questionnaire SF-36, which consists of a series of questions aimed at investigating eight aspects of physical and mental health; results expressed as a score ranging between 0 and 100, can be reported as two summary scores: the physical component summary (PCS) score and the mental component summary (MCS) score [88].

### 2.3. Dietary Protocols

The two study groups (KETO and MEDI) underwent, respectively, a VLCKD and a low-calorie MD protocol.

The MEDI group followed a traditional Mediterranean diet characterized by a macronutrient intake with a predilection for carbohydrate sources from wholegrain products and moderate consumption of proteins and fats [48]. The daily caloric intake was moderately low and was calculated on the estimated requirement, based on anthropometric measures, with a medium daily calorie deficit of 300–500 kcal, not less than the Resting Metabolic Rate (RMR).

The diet was structured with multiple isocaloric choices in the same food group, including protein, glycidic, and lipid sources in the three main meals and at least five daily servings of fruit and vegetables.

As regards the frequency of food consumption, no precise indications have been provided; however, it was advisable to vary the food choices, favoring fish, legumes, white meat, and extra-virgin olive oil as seasoning, typical of the Mediterranean style, and reduce the intake of red meat, full-fat dairy products, and refined foods. However, such products could be consumed as an exception to the diet.

The KETO group followed a multiphasic protocol, with exclusive initial use of protein meal replacement products (Therascience, Monaco, Principality of Monaco) and at least two daily servings of non-starchy vegetables. In addition, according to guidelines, omega-3, multivitamins, and multimineral supplementation were prescribed [76]. Each meal replacement product consisted of approximately 18 g of high-value proteins per serving; therefore, the number of products used daily was proportional to the protein requirement indicated by guidelines as 1.2–1.5 g/kg of ideal body weight [76]. This first phase (phase 1) lasted 30 days, while during the second and third phases, both lasting 15 days (phases 2 and 3), the first one and then two meals with natural protein foods (meat, fish, eggs) were inserted. After sixty days of ketosis, carbohydrates were gradually added, starting with fruit (phase 4); subsequently, every week (phases 5, 6, 7), a different food category containing carbohydrates (dairy products, legumes, cereals) was reintroduced, leading to a low-calorie Mediterranean diet, as that of MEDI group, in three months. The maintenance of the state of ketosis was ascertained by measuring blood levels of β-hydroxybutyrate (BOHB) through a finger-prick test and a handheld β-ketone analyzer (Menarini Areo, Florence, Italy). The test was performed before starting KD (T0) and during the sixty days of ketosis, specifically at T1 and T2.

### 2.4. Gut Microbiota Analysis

#### 2.4.1. Sample Collection

Stool samples from each subject were independently collected and transported to the laboratory within 3 h. Fresh samples were aliquoted and stored at −80 °C until further processing.

#### 2.4.2. Genomic DNA Extraction, Bacterial DNA Quantification, and 16S Libraries Preparation and Sequencing

Genomic DNA extraction from stool samples and quantification of bacterial DNA was performed as previously described [89]. The protocol of library preparation has been described in detail elsewhere [32]. 16S barcoded amplicon libraries were generated using primers targeting the V3-V4 hypervariable region of the bacterial 16S rRNA gene, and the Nextera XT index kit (Illumina, Inc., San Diego, CA, USA), and their size and quality were verified using D1000 reagents kit (Agilent Technologies, Santa Clara, CA, USA) on the Tapestation 4200 system (Agilent Technologies, Santa Clara, CA, USA). Amplicon libraries were quantified on the DeNovix QFX fluorometer by using the DeNovix dsDNA HS Assay Kit, normalized to a concentration equal to 4 nM, then pooled. Pooled libraries and the adapter-ligated library PhiX v3 used as a control were denatured and diluted to equal concentration (6 pM) and subsequently combined to obtain a PhiX concentration equal to 20% of the total volume. Combined 16S library and PhiX control were further denatured and sequenced on the MiSeq platform (Illumina) on a v3 paired-end run (300 × 2 cycles) using MiSeq v3 Reagent Kit (Illumina).

### 2.5. Bioinformatic and Statistical Analysis

Anthropometric, metabolic, lifestyle, and health status data, which represent continuous variables, are reported as Mean Value ± Standard Deviation (M ± SD). The comparison of data before and after NI in each group was performed using a t-test for paired data, while a t-test for independent samples was performed to compare the mean difference (T0-T3) of the same variables between the two groups; a *p*-value (*p*) < 0.05 was considered statistically significant.

As regards GM data, reads were processed using QIIME 2 [90]. First, primers were removed using cutadapt [91] (*q2-cutadapt* QIIME 2 plugin). Using DADA2 [92] (*q2-dada2* QIIME 2 plugin), reads were truncated at 3′ ends and filtered by quality. High-quality reads were denoised and merged to produce amplicon sequence variants (ASVs). Chimeric ASVs were filtered using VSEARCH [93] (*q2-vsearch* QIIME 2 plugin) and the SILVA database (v138) [94] as reference. Taxonomic assignment of ASVs was performed using a Naive-Bayes taxonomic classifier [95] (*q2-feature-classifier* QIIME 2 plugin) trained in a custom version of the SILVA database generated using RESCRIPt [96] (*q2-rescript* QIIME 2 plugin). Finally, non-bacterial ASVs (e.g., mitochondrial) were removed. The final taxonomic profiles were used as the input of PICRUSt2 [97] (*q2-picrust2* QIIME 2 plugin) to infer functional potential profiles from taxonomic data.

All microbiome analyses (plotting and statistics) were performed in R, and all plots were built with the R package *ggplot2* [98].

To account for different sequencing depths, samples were normalized to 22,173 reads (the lowest number of reads per sample) by Scaling with Ranked Subsampling (SRS) [99] using the SRS R package [100].

Alpha-diversity was assessed considering the ASV richness (observed ASVs) and the Shannon index, calculated with the R package *phyloseq* [101], and the Pielou’s J index, calculated with the R package *microbiome*, as alpha-diversity metrics. Alpha diversity differences between the two nutritional groups at each time point were assessed using the Mann–Whitney U test; alpha diversity differences across time points within each nutritional group were assessed using the Wilcoxon paired test.

Beta-diversity based on the Bray–Curtis distance matrix (calculated with the R package *phyloseq*), unweighted and weighted UniFrac metrics (calculated with the R package *rbiom* [102]) was compared between the two different nutritional groups at each time point and between each time point within the same nutritional intervention group using PCoAs and the PERMANOVA test.

The Generalized Linear Mixed-effects Model implemented in MaAsLin2 [103] was used to find significant longitudinal changes in bacterial taxa abundances (from phylum down to species level) during each diet. The patient was considered a random effect. The statistical significance was tested considering *p* ≤ 0.05, with a Benjamini–Hochberg (BH) correction cut-off at *q* ≤ 0.25.

## 3. Results

### 3.1. Anthropometric, Metabolic, Lifestyle, and Health Status Evaluation

Table 1 reports the anthropometric, metabolic, lifestyle, and health status data of the KETO and MEDI groups at baseline, which did not differ significantly from each other.

Table 2 and Table 3 show the anthropometric, metabolic, lifestyle, and health status data of KETO and MEDI groups before and after three months of NI. Both diets improved the anthropometric and metabolic status, mostly in the KETO group, which showed more significant results (Table 2). Patients following the VLCKD have achieved significant major progress in total weight reduction (−14.3 vs. −3.04 kg; *p* < 0.0001), BMI (−5.3 vs. −1.1 kg/m^2^; *p* < 0.0001), WC (−12.9 vs. −4.7 cm; *p* = 0.0006), and FM% (−7 vs. −3.1; *p* = 0.03) reduction, compared to MEDI group, while differences in FFM kg (−2.8 vs. +0.3; *p* = 0.053), FPG levels (−24.8 vs. +6.8 mg/dL; *p* = 0.08), HbA1c values (−1.15 vs. −0.7%; *p* = 0.45), lipid status, and BP values were not significant (Table 3). Moreover, a slight reduction and an increase, albeit not significant, of the phA were observed in the KETO and MEDI groups at T3, respectively; nonetheless, when the phA T0-T3 variation between the two groups was compared, a significant result was found (−0.2 vs. +0.3; *p* = 0.004).

Regarding the nutritional evaluation, both groups reduced the daily caloric intake and improved the adherence to MD, although that change did not reach a statistical significance; in addition, at T3, the KETO group significantly increased protein intake (19.7 ± 2.1% vs. 26.7 ± 2.6%; *p* = 0.004) and MDS scores relative to fish (1.7 ± 0.8 vs. 2.8 ± 0.7; *p*= 0.01) and vegetables (3.3 ± 1.5 vs. 5 ± 0; *p* = 0.04) intake. The detailed nutritional analysis results of both groups are reported in Supplementary Table S1 online.

The physical activity level and the daily sitting time remained unchanged in both groups. The SF-36 analysis showed a significant improvement of both PCS and MCS indicators, exclusively in the KETO group, while in the MEDI group, a slight decrease in both scores was observed (Table 2). Moreover, the SF-36 T0-T3 comparison between the two groups showed a significant result (Table 3).

Finally, the brief evaluation performed at T2, corresponding to the end of the ketosis phase of the VLCKD protocol, highlighted a significant reduction of body weight, BMI, and WC compared to baseline in the KETO group (*p* < 0.0001) and a significant reduction of WC (*p* = 0.004), but not of body weight and BMI in the MEDI group. Regarding these anthropometric variables, we also compared the results obtained at T2 and T3, observing a further significant reduction of body weight and BMI (*p* = 0.007), but not of WC in the KETO group, and a slightly significant reduction of BMI (*p* = 0.04) in the MEDI group.

As for the metabolic evaluation, the results of the auto-monitoring revealed an improvement of FPG exclusively in the KETO group, which at this time point showed evidence of the state of ketosis through the finger-prick test; moreover, one patient of this group reported the reduction of the anti-hypertensive therapy.

### 3.2. Gut Microbiota Analysis

**Figure 1:**
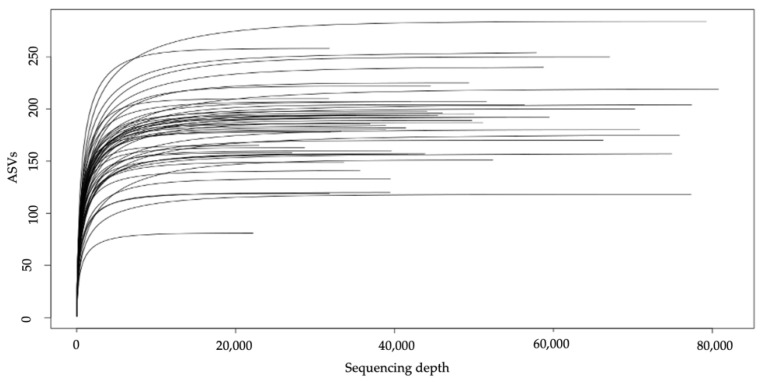
Amplicon sequence variants (ASVs) rarefaction curves per sample. Reads were selected by random subsampling without replacement at incremental steps of 50 reads.

First, the sequencing depth for each sample was assessed by calculating the Good’s coverage, which showed an excellent coverage (>99.993% for all samples). It has also been confirmed that all the microbiota diversity was sampled by drawing rarefaction curves, which showed that adequate sequencing depth was achieved (see Figure 1).

#### 3.2.1. Alpha and Beta Diversity Analysis

To account for different sequencing depths, samples were normalized to 22,173 reads (the lowest number of reads per sample).

**Figure 2:**
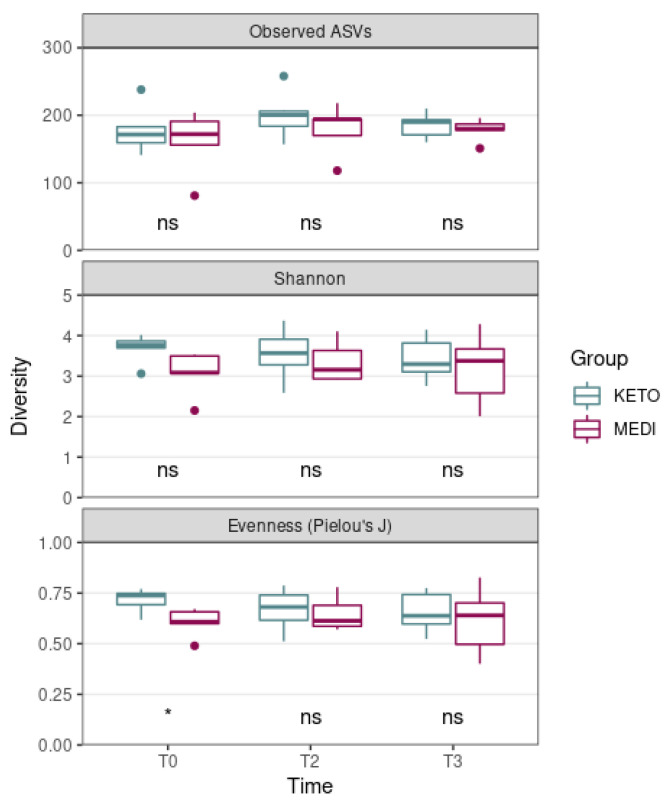
Gut microbiota alpha diversity comparison between diets at each time point. Each subplot concerns a different alpha diversity metric (Observed ASVs, Shannon, or Pielou’s J). The boxes highlight the median value and cover the 25th and 75th percentiles, with whiskers extending to the more extreme value within 1.5 times the length of the box. Statistical significance was evaluated by the Mann–Whitney U test, and it was indicated as follows: ns, non-significant; *, *p* ≤ 0.05. KETO = 6 patients who followed a very-low-calorie ketogenic diet (VLCKD), MEDI = 5 patients who followed a low-calorie Mediterranean diet (MD).

The Mann–Whitney U test showed no statistically significant differences in the ASV richness and in the Shannon index across the two nutritional groups over time, while the Pielou’s J index, at baseline, was significantly higher in KETO, compared to MEDI (*p* = 0.017, see Figure 2 and Supplementary Table S2 online).

**Figure 3:**
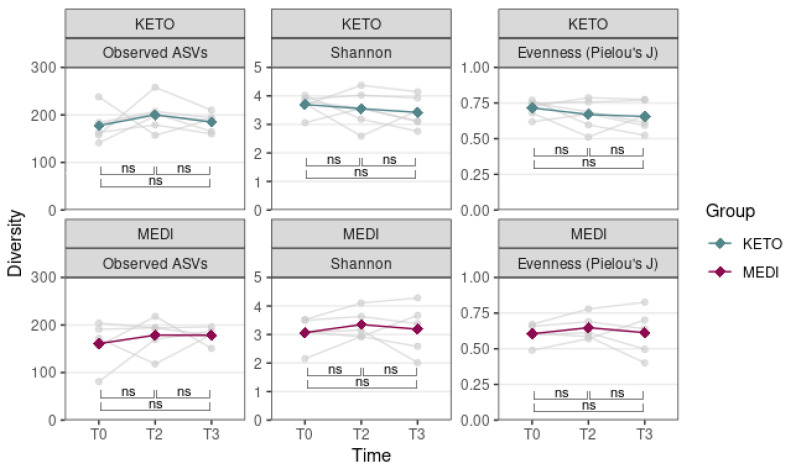
Gut microbiota alpha diversity comparison between time points for each diet. Each subplot concerns a different combination of alpha diversity metric (Observed ASVs, Shannon, or Pielou’s J) and diet (KETO or MEDI). Samples from the same patient are linked by a gray line. The colored lines depict the mean values at each timepoint. Statistical significance was evaluated by the paired Wilcoxon signed-rank test, and it was indicated as follows: ns, non-significant. KETO = 6 patients who followed a very-low-calorie ketogenic diet (VLCKD), MEDI = 5 patients who followed a low-calorie Mediterranean diet (MD). Samples were analyzed at baseline (T0), after two months (T2), and after three months (T3) of nutritional intervention.

Moreover, no statistically significant differences in all alpha diversity indices throughout the duration of each nutritional intervention were observed, which was assessed by performing a Wilcoxon paired test between different time points (see Figure 3 and Supplementary Tables S3 and S4 online).

**Figure 4:**
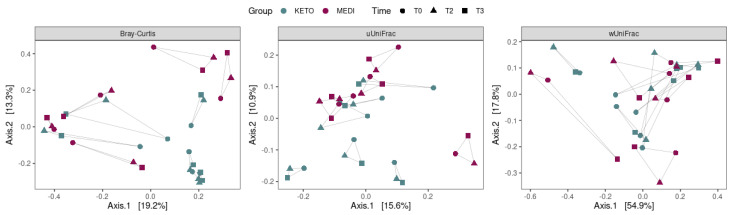
Principal coordinate analysis plots showing gut microbiota compositional changes along time points for each diet. Each subplot concerns a different beta diversity metric (Bray-Curtis, unweighted UniFrac, or weighted UniFrac). Samples from the same patient are linked by a gray line. KETO = 6 patients who followed a very-low-calorie ketogenic diet (VLCKD), MEDI = 5 patients who followed a low-calorie Mediterranean diet (MD). Samples were analyzed at baseline (T0), after two months (T2), and after three months (T3) of nutritional intervention.

Concerning the beta diversity, the compositional trajectory of each patient along the nutritional intervention is shown in Figure 4.

**Figure 5:**
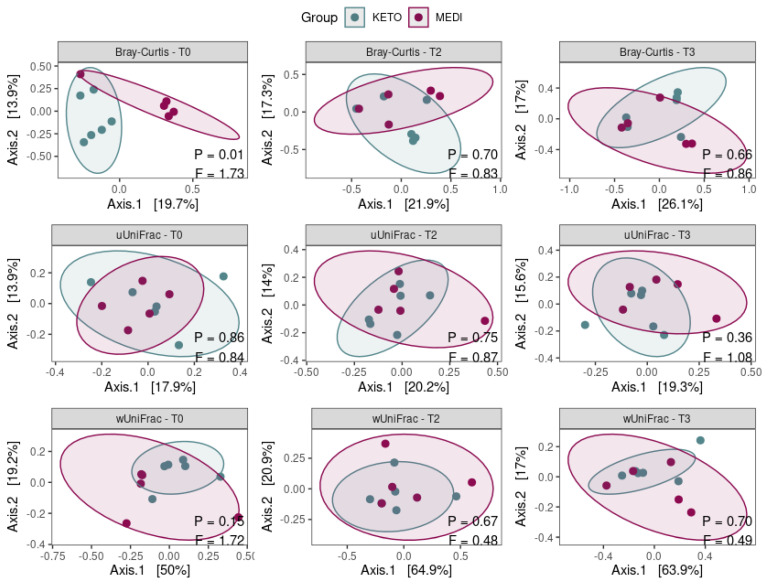
Principal coordinate analysis plots with comparisons of gut microbiota composition between diets at each time point. Each subplot concerns a combination of beta diversity metric (Bray-Curtis, unweighted UniFrac, or weighted UniFrac) and timepoint (T0, T2, or T3). Ellipsoids depict the 90% compositional confidence interval. Statistical significance was evaluated by the PERMANOVA test, with statistical summaries included in each subplot. KETO = 6 patients who followed a very-low-calorie ketogenic diet (VLCKD), MEDI = 5 patients who followed a low-calorie Mediterranean diet (MD).

The Principal Coordinates Analysis (PCoA) based on the Bray-Curtis dissimilarity matrix showed a marked separation between the GM communities of KETO and MEDI at baseline (see Figure 5 and Supplementary Table S5 online), confirmed by PERMANOVA analysis, which indicated a significant difference in beta diversity between cohorts (sum of squares = 0.599, mean of squares = 0.599, F = 1.727, R^2^ = 0.161, *p* = 0.013). However, the beta diversity based on the unweighted and weighted UniFrac metrics across the two nutritional groups did not show any statistically significant difference over time, as shown in Figure 5 and in Supplementary Table S5 online.

**Figure 6:**
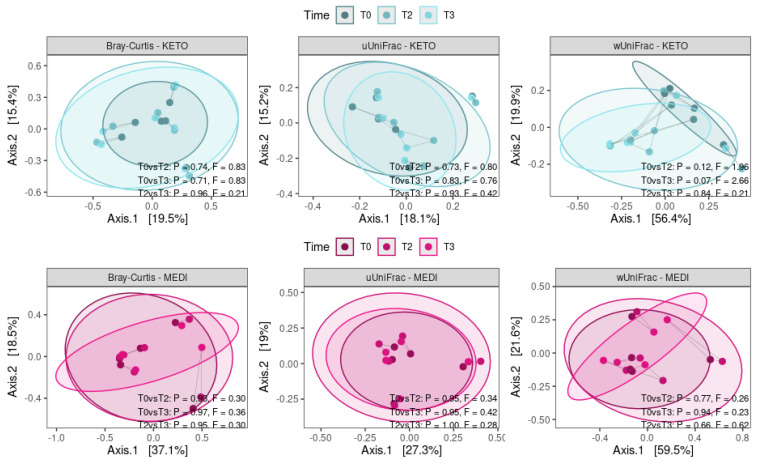
Principal coordinate analysis plots with comparisons of gut microbiota composition between time points for each diet. Each subplot concerns a combination of beta diversity metric (Bray-Curtis, unweighted UniFrac, or weighted UniFrac) and diet (KETO or MEDI). Ellipsoids depict the 90% compositional confidence interval. Samples from the same patient are linked by a gray line. Statistical significance was evaluated by the PERMANOVA test, with statistical summaries included in each subplot. KETO = 6 patients who followed a very-low-calorie ketogenic diet (VLCKD), MEDI = 5 patients who followed a low-calorie Mediterranean diet (MD). Samples were analyzed at baseline (T0), after two months (T2), and after three months (T3) of nutritional intervention.

Similarly, no statistically significant differences in beta diversity throughout each nutritional intervention were obtained by performing a pairwise analysis between each time point (see Figure 6 and Supplementary Tables S6 and S7 online).

#### 3.2.2. Compositional Analysis of Intestinal Microbiota

**Figure 7:**
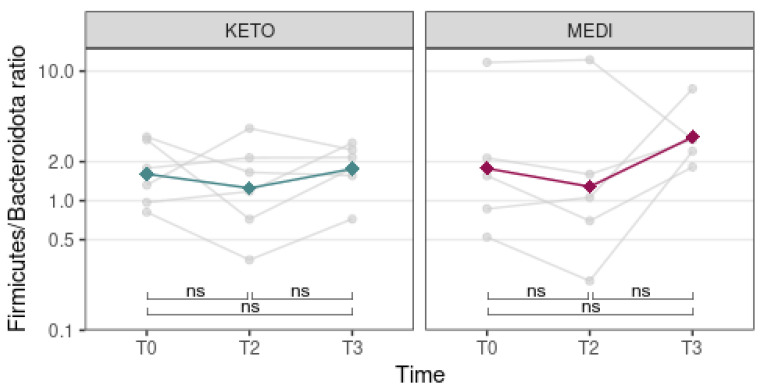
Gut microbiota Firmicutes/Bacteoroidota ratio comparison between time points for each diet. Each subplot concerns a different diet (KETO or MEDI). Samples from the same patient are linked by a gray line. The colored lines depict the mean values at each time point. A log_10_ y-axis was used. Statistical significance was evaluated by the paired Wilcoxon signed-rank test, and it was indicated as follows: ns, non-significant. KETO = 6 patients who followed a very-low-calorie ketogenic diet (VLCKD), MEDI = 5 patients who followed a low-calorie Mediterranean diet (MD). Samples were analyzed at baseline (T0), after two months (T2), and after three months (T3) of nutritional intervention.

Although some volunteers presented huge variations in the Firmicutes/Bacteroidota ratio during the nutritional intervention, no statistically significant differences were detected between timepoints in both diet groups (see Figure 7 and Supplementary Tables S8 and S9 online).

**Figure 8:**
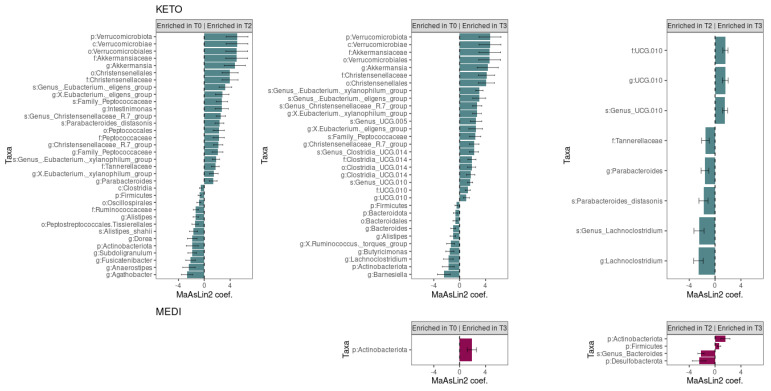
Changes in gut microbiota taxa abundances between time points for each diet. Each subplot concerns a comparison between timepoints (T0 vs. T2, T0 vs. T3, or T2 vs. T3) in one of the diets (KETO, green MEDI, purple). Statistical significance was evaluated by running a Generalized Linear Mixed-effects Model with MaAsLin2. Effect size is represented by the MaAsLin2 model coefficients and respective standard errors. Only taxa abundance changes at *p* ≤ 0.05 and *q* ≤ 0.25 are considered statistically significant. *q*: *p* adjusted for Benjamini–Hochberg (BH) correction test with a cut-off at *q* ≤ 0.25. KETO = 6 patients who followed a very-low-calorie ketogenic diet (VLCKD), MEDI = 5 patients who followed a low-calorie Mediterranean diet (MD). Samples were analyzed at baseline (T0), after two months (T2), and after three months (T3) of nutritional intervention.

The Generalized Linear Mixed-effects Model, confirmed after multiple testing corrections, with a cut-off at *q* ≤ 0.25, showed several significant microbial markers associated with the nutritional intervention, almost exclusively with the ketogenic one (see Figure 8). Specifically, we performed a pairwise analysis between each GM community time point within the specific study group.

As regards the KETO group, results showed that after two months of dietary protocol, twenty-one bacterial taxa significantly increased, while thirteen were significantly reduced; after three months of NI, twenty-two microbial taxa were significantly elevated, and ten were significantly depleted. Moreover, the comparison between GM communities at T2 and T3 time points showed that three taxa were enriched in T3 and five taxa were enriched in T2 (see Figure 8 and Table 4). Results were ranked by their MaAsLin2 coefficient: the Verrucomicrobiota phylum was identified as the main biomarker in KETO, together with its members Verrucomicrobiae, Verrucomicrobiales, Akkermansiaceae, and *Akkermansia,* both at T2 and T3 of nutritional intervention; while within the Firmicutes phylum the strongest associations were related to Christensenellales order and Christensenellaceae family in the same time points. At the same time, the Actinobacteroidota phylum was significantly depleted both at T2 and T3; while, within the Firmicutes phylum, genera belonging to Lachnospiraceae family (*Agathobacter*, *Anaerostipes*, *Fusicatenibacter*, and *Dorea*), to Ruminococcaceae family (*Subdoligranulum*) were significantly depleted as a consequence of two months of NI in KETO and the *Barnesiella* and *Butyricimonas* genera (belonging to Bacteroidota phylum and Bacteroidales order), the *Lachnoclostridium* and *X Ruminococcus torques group* genera (belonging to Firmicutes phylum and Lachnospiraceae family) were significantly reduced after three months of NI in the same patients compared with baseline. Furthermore, the UCG 010 family and its unclassified members at the genus and species level showed a strong association in KETO as a consequence of the NI from T2 to T3; by contrast, the genus *Lachnoclostridium* and a *Lachnoclostridium* unclassified species (belonging to Firmicutes phylum), the Tannerellaceae family and its members *Parabacteroides* and *Parabacteroides distasonis* (Bacteroidota phylum) were significantly associated at T2 compared with T3 (see Figure 8 and Table 4).

As regards the MEDI group, we observed that no taxa varied significantly after two months of the dietary protocol. The Actinobacteroidota phylum was identified as the only taxon that increased after three months of NI compared to baseline; while, by comparing the GM communities of T2 and T3 time points, in addition to Actinobacteroidota, also strong associations were related to Firmicutes phylum at T3. The Desulfobacterota phylum and a species belonging to the genus Bacteroides were significantly associated with T2 time points compared to T3 in the same patients (see Figure 8 and Table 5).

**Figure 9:**
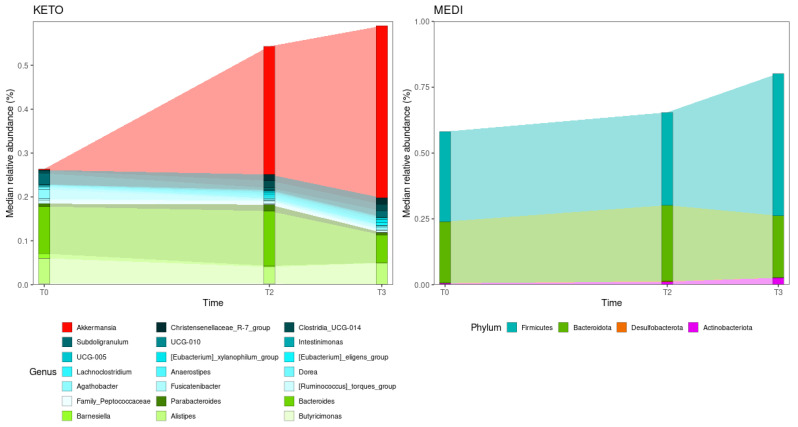
Relative abundance changes in gut microbiota taxa between time points for each diet. Only taxa significantly enriched or depleted during the nutritional intervention (according to MaAsLin2 models) are shown (at the genus level for KETO and at the phylum level for MEDI). Genera are colored in the KETO plot based on the phylum to which each genus belongs (red: Verrucomicrobia; blue: Firmicutes; green: Bacteroidota). KETO = 6 patients who followed a very-low-calorie ketogenic diet (VLCKD), MEDI = 5 patients who followed a low-calorie Mediterranean diet (MD). Samples were analyzed at baseline (T0), after two months (T2), and after three months (T3) of nutritional intervention.

Abundance changes between time points can be further contemplated by representing the relative abundance fluctuations along the nutritional intervention (see Figure 9). Because most MaAsLin2 associations for KETO occur at the genus level and most MaAsLin2 associations for MEDI occur at the phylum level, Figure 9 depicts significantly enriched or depleted taxa at the genus and phylum levels for KETO and MEDI, respectively.

#### 3.2.3. Functional Metagenome Prediction Analysis

**Figure 10:**
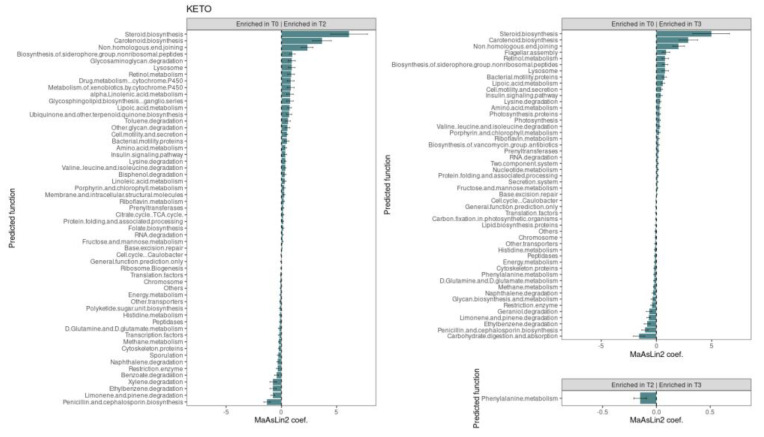
Changes in gut microbiota predicted function abundances between time points for each diet. Each subplot concerns a comparison between time points (T0 vs. T2, T0 vs. T3, or T2 vs. T3). Statistical significance was evaluated by running a Generalized Linear Mixed-effects Model with MaAsLin2. Effect size is represented by the MaAsLin2 model coefficients and respective standard errors. Only predicted function abundance changes at *p* ≤ 0.05 and *q* ≤ 0.25 are considered statistically significant. KETO = 6 patients who followed a very-low-calorie ketogenic diet (VLCKD). Samples were analyzed at baseline (T0), after two months (T2), and after three months (T3) of nutritional intervention.

Comparative prediction analysis of the functional metagenome was performed using PICRUSt2. A total of seventy significant metabolic pathways were identified in KETO over time (see Figure 10 and Supplementary Table S10 online). In particular, the common twenty-two pathways were significantly increased both at T2 and T3 compared with baseline, while the common seventeen pathways were significantly reduced at the same time points. Of the remaining thirty-one pathways, eleven and six were significantly increased and reduced, respectively, after two months of NI and, similarly, after three months, although with small effect size; seven and six were significantly increased and reduced, respectively, after three months of NI and, at the same time, after two months, although with small effect-size. In addition, only one pathway (phenylalanine metabolism) significantly decreased at T3 compared with T2, consistently with a reduction at T2 and T3 compared with baseline, although with a small effect size.

In KETO, the strongest associations were positively related to steroid biosynthesis, carotenoid biosynthesis, and non-homologous end-joining pathways both at T2 and T3 compared with baseline, while penicillin and cephalosporin biosynthesis, limonene, and pinene degradation and ethylbenzene degradation pathways were strongly and negatively associated with the same time points. Moreover, among other strongly associated pathways, xylene degradation was significantly and negatively associated at T2, while at T3, it was reduced, although with a small effect size; carbohydrate digestion and absorption were the most strongly and negatively associated pathway with T3 compared with baseline, while at T2 it was reduced, although with small effect-size. No pathway was significantly associated with MEDI over time.

## 4. Discussion

This study evaluated the short-term impact of two dietary models (VLCKD and MD) on the GM and its functional profile of eleven patients with overweight or obesity, recently diagnosed with T2DM; among them, six subjects followed the VLCKD (KETO group) [76], while five subjects followed the low-calorie MD (MEDI group) [46]. At baseline and after 3 months of NI, all patients underwent a multidimensional evaluation including anthropometric, clinical, metabolic, lifestyle, and quality of life assessment, while the GM evaluation was also performed after 2 months of NI along with a brief anthropometric assessment.

Lifestyle interventions are an essential element in the treatment of patients with T2DM, overweight or obesity, and metabolic alterations, especially in the initial phase of T2DM, with the aim of losing weight, reducing visceral fat, and managing the disease without drugs [39,104]. Nonetheless, it remains to be ascertained which type of physical activity to be practiced, the best dietary protocol to be followed, and whether the improvement in the metabolic profile is to be attributed to weight loss, regardless of the proposed dietary approach.

Several randomized clinical trials have shown that an increase in physical activity associated with proper nutritional education allows a significant reduction in body weight and an improvement in blood pressure, lipid profile, and glycemic control in elderly patients with T2DM [105,106], supporting the key role of regular practice of physical activity to preserve muscle and bone mass during weight loss, especially in such kind of patients [107]. On the other hand, adapted programs, including different types of moderate exercise not associated with a nutritional intervention [108], although they have been shown to improve overall physical and psychological health [109,110,111], were not able to reduce body weight and to improve dysmetabolism in older adults with overweight [112].

As for nutrition, MD, in its different variations, is commonly considered the best way to manage T2DM [46] due to the simplicity and frugality of the dishes it promotes, its versatility, the possibility of sporadically indulging in extra foods, its richness in healthy nutrients paired with a low caloric density, and its support to the microbiota [24,113,114]. In this regard, a recent study found that in nine patients with T2DM, the increased adherence to MD after a three months NI resulted in beneficial changes in the GM profile, which seemed to precede the FPG and HOMA index reduction, hypothesizing a relevant role of the diet-related GM modulation on the metabolic improvement [115].

Nonetheless, new dietary approaches with lower carbohydrate quantities, such as VLCKD, could be used to manage carbohydrate dysmetabolism better and promote greater weight loss even in the long term [73], but there are still little data concerning the effects of VLCKD, especially in T2DM patients, on the GM. On this issue, the Firmicutes/Bacteroidetes ratio that often characterizes the microbiota of obese patients [33] has shown only a slight modification after a VLCKD, while plasma metabolome and fecal bile acid composition present larger variations [116].

A study by Gutierrez-Repiso et al. [117] has compared the MD, KD, and Bariatric Surgery (BS; in this case, sleeve gastrectomy), showing that microbiota variations were related to the approach used. In particular, MD increased the activity of Short-Chain Fatty Acid (SCFA) producers, also known as metabolic regulators, while KD and BS presented common traits, such as *Lactobacillus* reduction. The effects of *Lactobacillus* on obesity seem to be species-dependent, i.e., some strains are linked to weight gain, others to weight loss [118].

Furthermore, a recent review by Rondanelli et al. has analyzed various types of KD [116], finding a reduction in SCFA production and, among bacteria, the reduction of *Roseburia* and *Eubacterium rectale*, the two main producers of butyrate. In addition, an increase in *Christensenellaceae* and *Akkermansia* was also observed, while for *F. prausnitzii*, usually considered a marker of good health, data were mixed, and its low levels could be linked to the presence of T2DM [119].

In the same review, the analysis of the microbiota composition during the different phases of KD showed an initial reduction in *Bifidobacteria* and *Lactobacillus* due to the lack of substrate to use, i.e., cereal fibers, and an increase in *Bacteroides*, probably due to the prevalence of proteins. Nonetheless, if proteins of vegetable origin are used, the increase in *Bacteroides* is reduced [120]. On the other hand, in the phases of reintroduction of carbohydrate sources, recovery of *Bifidobacteria* and *Lactobacillus* was observed, suggesting that these changes are transient and more linked to the composition of diet than to weight loss. The reduction of *Bifidobacteria* may also promote a reduction of Th-17 cells, whose activation is associated with autoimmune diseases [121]. Other species that tend to increase are *Alistipes* and *Parabacteroides* [116].

To avoid damaging the microbiota and losing friendly species during KD, the review by Paoli et al. recommends using fermented foods which do not interfere with ketosis, the use of prebiotics and probiotics, a correct balance between omega 3 and omega 6 fatty acids, and the presence of sources of MUFA, such as olive oil. In addition, it suggests the restriction of animal proteins and the use of meal replacements, including vegetable proteins, such as pea-derived ones, along with whey proteins [122].

Recently, a trial showed that the administration of a symbiotic, formed by Bifidobacteria and a prebiotic fiber, can improve weight loss and metabolic state during VLCKD by working on the microbiota with consequent reduction of inflammation. The influence on GM was apparently minimal, but SCFA production was markedly increased [123]. This finding highlights that the use of probiotics and/or prebiotics could be encouraged in VLCKDs and, in general, in slimming diets [124]. Avoiding sweeteners and particularly using the galacto-oligosaccharides and fructo-oligosaccharides bifidogenic fibers can further contribute to the health of the microbiota [122].

In our study, after three months of NI, both groups of patients had a significant weight and BMI loss, but patients subjected to VLCKD showed a significantly higher reduction in body weight, BMI, WC, and FM, compared to those following the MD, without any significant difference in FFM variations. The slight difference observed in the variation of the phA between the KETO and MEDI groups in the third month of NI could be attributed to slight dehydration and a reduction of cell metabolism in the KETO group. In fact, the phA is known as a marker of cell integrity, and its association with good adherence to MD has been reported [54]. On the other hand, the metabolic status improved similarly in both groups. In particular, the HbA1c value, a marker of diabetes glycometabolic control, showed a more significant decrease in the KETO group after three months of NI; however, the comparison of variations obtained at T3 between the two groups did not show any significant difference. This finding is similar to the result obtained by Gardner et al., who compared the metabolic effects of a well-formulated KD and an MD in a study including patients with pre-diabetes and T2DM. However, these authors did not report any significant difference in weight loss between the two diets [125]. As regards weight loss, our results partly confirm those of Moriconi et al., who reported a statistically significant BMI reduction exclusively in patients with T2DM and obesity following a three months VLCKD compared to a control group following a standard low-calorie diet (LCD) [126]. In this study, VLCKD, but not LCD, was also associated with a significant reduction of HbA1c values and a significant improvement in eating patterns and quality of life [126].

In our patients, the lifestyle evaluation highlighted an improvement in eating habits in both groups, with an increase in adherence to MD not only in the MEDI group, according to expectations, but also in the KETO group, which significantly increased the daily intake of fish and vegetables. However, it should be pointed out that the follow-up assessment was performed after three months of NI when both groups were following a similar dietary protocol based on MD principles. This result is in line with the study of Landry et al., who found a similar mean adherence between ketogenic and Mediterranean diets among patients with prediabetes or T2DM [127]. On the contrary, the mean value of physical activity level did not vary during the intervention period, although, considering individual data, four out of eleven patients enrolled (two in each group) who were sedentary at baseline became moderately active at T3. In this regard, it should be emphasized that most of the data were collected during the lockdown period in Italy (March–May 2020), with obvious logistical difficulties and complications for the subjects of the study to carry out structured and outdoor physical activity. Moreover, unlike for NI, for physical activity, no specific indications, but only general recommendations, have been given, and the assessment was performed based on self-reported data using the IPAQ but not objectively measured [128].

Furthermore, the KETO group showed a significant improvement in both physical and mental synthetic scores on the quality of life questionnaire, while the MEDI group showed a slight reduction. The comparison of the variations between the two groups at T3 was also significant. This result could be related to an improved perceived mood and energy level previously described after a low-glucose, not ketogenic diet compared to a high-glucose [129], and, limited to PCS, also reported in women affected by ovarian or endometrial cancer after 12 weeks of KD [130]. Moreover, a significant improvement in physical and mental health scores of the SF-36 questionnaire was found in diabetic patients subjected to VLCKD in the aforementioned study by Moriconi et al. [126].

Concerning the short-term impact of the two dietary models on GM, we first evaluated whether the gut microbial community was different, at baseline, between the two study groups by evaluating alpha and beta diversity. Specifically, we observed a significantly greater evenness of the microbial structure in the KETO group than in the MEDI group at baseline, although significance was lost over time. However, neither the richness of the number of species nor the Shannon index (a mathematical expression that combines species richness and evenness as a measure of alpha diversity) was significantly different between the two study groups. As regards the beta diversity, PERMANOVA analysis indicated a significant difference based on the Bray-Curtis distance matrix between the GM communities of KETO and MEDI only at baseline, although the beta diversity based on the unweighted and weighted UniFrac metrics across the two nutritional groups did not show any statistically significant difference at baseline and over time. Nonetheless, it should be pointed out that at baseline, there was no significant difference in anthropometric, clinical, or lifestyle variables between the two study groups.

No statistically significant differences in all alpha and beta diversity indices throughout treatments were observed. It should be emphasized that although significant differences are found at higher microbiome resolution, these are not always confirmed by differences in summary metrics such as alpha and beta diversity, as observed after NI on other study cohorts [60,131].

Although some volunteers presented huge variations in the Firmicutes/Bacteroidota ratio along the NI, no statistically significant differences between time points in both diet groups were detected.

By deepening GM characterization through taxonomic analysis, by means of the Generalized Linear Mixed-effects Model, confirmed after multiple testing corrections, with a cut-off at *q* ≤ 0.25, we observed several significant microbial markers associated with the NI and, but almost exclusively with the ketogenic one. Results were ranked by their MaAsLin2 coefficient: the Verrucomicrobiota phylum was identified as the main biomarker in KETO, together with its members Verrucomicrobiae, Verrucomicrobiales, Akkermansiaceae, and *Akkermansia,* both at T2 and T3 of NI. Interestingly, these beneficial taxa showed a significant increase up to three months of NI in KETO but not in MEDI, although in the former, the NI at the end of phase T3 corresponded to the low-calorie MD of the MEDI group, whereas the T2 of KETO corresponded to the end of the ketosis phases. *Akkermansia muciniphila* represents the most studied microorganism belonging to these taxa, which is considered a significant biomarker of intestinal homeostasis, whose physiological effects in promoting intestinal integrity for its capacity to stimulate the mucous turnover rate are well documented [132,133]. *A. muciniphila* contributes to intestinal health and glucose homeostasis [132,134] and has been shown to improve the metabolic status and clinical outcomes after a dietary intervention in overweight/obese adults [135], and have protective effects on diet-induced obesity [136,137]. Moreover, it has been proposed to regulate adipose tissue metabolism and the accumulation of fat [138] and its increase has been associated with KD [116]. *A. muciniphila* supplementation in patients with overweight/obesity was associated with reduced inflammation marker levels and improved several metabolic parameters [139], while in animal models of diabetes and obesity restored the integrity of the epithelial mucosa, improved glucose tolerance, and metabolic parameters [140]. Contrariwise, its depletion has been associated with many diseases, such as inflammatory bowel disease and metabolic disorders [141]. As confirmation of their beneficial effect, the Verrucomicrobia phylum, together with its members Verrucomicrobiaceae, *Akkermansia*, and *Akkermansia muciniphila,* was identified as the main biomarker in centenarian subjects [32,142,143,144]. Within the Firmicutes phylum, the strongest associations were also related to the Christensenellales order and Christensenellaceae family both at T2 and T3 of VLCKD, as previously described [116]. An unclassified genus and species from Christensenellaceae_R.7_group were also strongly associated, albeit with a lower MaAsLin2 coefficient.

This constitutes an interesting finding on the impact of the ketogenic diet on obese patients with T2DM, considering that a reduction in Christensenellaceae was observed in individuals with pre-type 2 diabetes [145]. Christensenellaceae are involved in the fermentation of proteins and fibers and have been associated with a diet low in refined sugars and high in fruit and vegetables, with a consumption of dairy products and an increase in animal products in the diet [146]. These pieces of evidence are consistent with a protein and non-starchy vegetable intake in the first sixty days of the ketogenic diet in KETO, followed by the gradual introduction of fruit, dairy products, legumes, and cereals until T3. Furthermore, Goodrich et al. showed that the inoculation of the obese human microbiome in germ-free mice fed a high-fiber diet induced a reduction in adiposity only in mice receiving fecal transplant modified with the addition of *Christensenella minuta*, compared to those receiving unmodified stools or stools containing non-viable *C. minuta* [147].

Interestingly, the Christensenellaceae family has been associated with a lean phenotype, negatively correlated with visceral fat mass, trunk fat, android fat [102], waist circumference, and waist/hip ratio [146]; and it is increased after a reduction in body weight in obese postmenopausal women following NI [148]. These data are consistent with a significantly greater reduction in body weight, BMI, WC, and FM in our KETO cohort compared to MEDI, in which no significant increase in these bacteria taxa was observed.

The Christensenellaceae family has also been negatively associated with dyslipidemia [149,150] and positively associated with healthy glucose metabolism [151,152]. Consistently, fasting blood glucose showed a greater improvement, although not significant, in the KETO group than in MEDI; moreover, as regards the value of HbA1c, a more significant decrease was reported in the KETO group after three months of NI. Similarly to the Verrucomicrobia phylum and its members, Christensenellaceae has also been associated with human longevity [142,144,153,154], being a marker of human health.

In the KETO group, the T2 and T3 time points also shared the increase, within the Firmicutes phylum, in some taxa belonging to the Lachnospiraceae family (the unclassified species from *Eubacterium xylanophilum* group and *Eubacterium eligens* group, and their related genera) and in the unclassified Family Peptococcaceae species. In line with the typical dietary regimen of our cohorts, the presence of *Eubacterium* spp. in the gut has been associated with increased intake of dietary fibers, as several species are able to utilize digestion-resistant complex carbohydrates [155]. In addition, the abundance of *Eubacterium* spp. can be positively modulated following a diet rich in omega-3 polyunsaturated fatty acids, which our KETO patients took as a supplement [155]. For their beneficial implications, such as the modulation of gut inflammation through SCFAs, multiple species of the *Eubacterium* genus are currently considered promising targets for therapeutic strategies.

As regards the Peptococcaceae family, Sha Di et al. in 2019 observed that fatty acids produced by many bacteria, such as the Peptococcaceae family, are involved in the regulation of glucose homeostasis, lipid metabolism, and insulin sensitivity, as well as choline, produced by the same family [156]. Previous studies also found that fasting serum levels of glycerol, monounsaturated fatty acids, and saturated fatty acids are strongly associated with a lower abundance of Peptococcaceae, while polyunsaturated fatty acids, including omega-6, docosahexaenoic acid (DHA), omega 3 and linoleic acid are positively associated [145]. Furthermore, the role of Peptococcaceae has yet to be defined, given that they have been negatively associated with pathological conditions [157] but also identified as biomarkers of the chronic progressive disease course of experimental autoimmune encephalitis (CP-EAE) [158].

The T2 and T3 time points also share a significant reduction in Firmicutes and Actinobacteriota phyla and in the *Alistipes* genus (Bacteroidota phylum). The reduction in Firmicutes is not surprising, considering that this phylum was significantly increased in obesity, as well as several of its members that express propionate production pathways [159,160], and was reduced in obese patients following a moderately hypocaloric MD [60]. With regards to the Actinobacteria phylum, it was found to be elevated in obese patients [32,161], while *Alistipes* is implicated in colorectal cancer and associated with depression and inflammation [162].

With reference only to the ketosis phase, therefore to the T2 time point, we observed the reduction in several taxa belonging to the Firmicutes phylum and, in particular, the Clostridia class, the order Peptostreptococcales, Tissierellales, and the order Oscillospirales, together with its members Ruminococcaceae family (and its *Subdoligranulum* genus) and Lachnospiraceae family (and its *Anaerostipes*, *Dorea*, *Agathobacter*, and *Fusicatenibacter* genera). The *Alistipes shahii* species belonging to the Bacteroidota phylum was also reduced. The reduction in members of the Lachnospiraceae family following KD is not surprising, considering that it was also observed as a consequence of other hypocaloric diets (high protein, fiber-rich, or with prebiotic supplementation) [60,159,160,163]. Members of this family can hydrolyze starch and other sugars to produce butyrate and other SCFAs and play a central role in the mechanisms of bacterial cross-feeding [164]. Although Lachnospiraceae are known as beneficial bacteria, their increased proliferation has been associated with metabolic diseases in both human and animal studies and with obesity [32]. At the same time, higher SCFA production (acetate, propionate, and butyrate) was associated with intestinal dysbiosis and obesity [165,166], and emerging evidence indicates the pathological effects, in various disorders, including obesity, of specific SCFA, such as acetate and propionate [167,168].

As regards *Subdoligranulum*, higher abundances were observed in GIT neoplasms [169], and its association with chronic inflammation and poor metabolic control [170], with blood markers of inflammation and endotoxemia in Type 1 diabetes mellitus (T1DM) [171] has been demonstrated.

At the end of the ketosis phase, the *Intestinimonas* genus belonging to the Oscillospiraceae family (Firmicutes phylum) and the Peptococcales order with its members Peptococcaceae and an unclassified genus from Peptococcaceae family (Firmicutes phylum) were increased. Interestingly, *Intestinimonas* was found to be elevated in diabetic models of mice undergoing treatment with Lycium barbarum (LBP), a polysaccharide used to alleviate T2DM through the modulation of intestinal microbiota [172]. Moreover, in Swedish subjects naive for diabetes treatment and grouped by glycemic status, *Intestinimonas* was found to be depleted in the group with impaired fasting glucose (IFG) [173], while an increased abundance of *Intestinimonas butyriciproducens* and *Akkermansia muciniphila* has been reported, together with improvements in glucose and insulin sensitivity, in high cardiometabolic risk subjects following an MD [174,175].

A further result of our research was the strong association at T3 in the KETO group of the UCG 010 family and its unclassified members at genus and species level (Firmicutes phylum) as a consequence of the NI from T2 to T3 and from T0 to T3. It should be noted that these taxa, whose physiological role remains to be defined, were not significantly increased at T2 compared to T0; therefore, they were associated with the MD only after following the KD.

*Lachnoclostridium* was found to be depleted at the end of T3, both in T3 *versus* T0 and in T3 *versus* T2, but not in T2 compared to T0. Interestingly, despite *Lachnoclostridium* harboring species that produce SCFAs, mainly butyrate, to which favorable immunomodulating actions are ascribed [176,177], its reduction was observed in Diabetes-Induced Cognitive Impairment (DCI) models following the treatment with the bioactive compound tanshinone IIA (TAN) [178], or in mice with T2DM following the anti-hyperglycemic treatment [179].

Concerning the Tannerellaceae family and its members, *Parabacteroides* and *Parabacteroides distasonis* (Bacteroidota phylum), we have observed an inconsistent trend throughout the NI in the KETO group. In particular, these taxa increased at T2 compared with baseline and decreased at T3 compared with T2, without significant variation between T0 and T3, suggesting that their increase is strictly associated with the purely ketogenic diet. A significant decrease in the relative abundance of *Parabacteroides* genus and *P. distasonis* species was observed in Sardinian obese patients [32], consistent with what was previously observed by Del Chierico et al., which have associated both *Parabacteroides* and *Parabacteroides distasonis* with normal body weight [180]. The metabolic benefits of *Parabacteroides distasonis* on decreasing weight gain, hyperglycemia, and hepatic steatosis in ob/ob and high-fat diet (HFD)-fed mice have also been reported [181], although its administration has been shown to induce depressive-like behavior in mouse models [182].

Our research highlighted the significant change in some taxa limited to the comparison between T3 and baseline in the KETO group. Specifically, the increase in the order Clostridia UCG.014 and its unclassified family, genus, and species taxa, together with the increase in the unclassified Genus UCG.005 species belonging to the family Oscillospiraceae was observed; whereas the reduction in the *X.Ruminococcus._torques_group* genus (Firmicutes) and Bacteroidota phylum together with several of its members, such as the Bacteroidales order and the Bacteroides (Bacteroidaceae), Barnesiella (Barnesiellaceae) and Butyricimonas (Marinifilaceae) genera has also been observed. The biological relevance of the increase in Clostridia UCG.014 needs further investigation, as a study on the effect of berberine on hyperglycemia and gut microbiota composition in type 2 diabetic Goto-Kakizaki rats showed a decrease [183]. On the other hand, to support our findings, Clostridia_UCG-014 was found to be significantly elevated as a consequence of physical activity in more active older adults with insomnia and positively correlated with physical activity levels [184]. It should be pointed out that despite the mean value of physical activity level did not vary during the intervention period in our study, four out of twelve patients enrolled (two in each study group) who were sedentary at baseline became moderately active at T3.

The *Ruminococcus torques* group significantly decreased at T3 in our KETO group, which is thought to be a detrimental factor in diabetic nephropathy together with the genera *Alistipes*, *Bacteroides*, *Subdoligranulum*, and *Lachnoclostridium* [185]. In addition, *Ruminococcus torques* decreased after bariatric surgery and diabetes remission [186].

As regards the MEDI group, we observed that no taxa varied significantly after two months of the dietary protocol. The Actinobacteroidota phylum was identified as the only biomarker after three months of NI; while, by comparing the GM communities of T2 and T3 time points, in addition to Actinobacteroidota, strong associations were also related to the Firmicutes phylum at T3 and to Desulfobacterota phylum and an unclassified species from the *Bacteroides* genus at T2. A recent systematic review of the changes in intestinal microbial profiles caused by T2DM treatment [187] identified an increase in Actinobacteria following Roux-Y gastric bypass (RYGB) [186,188]. Furthermore, an increase in Actinobacteria concurrently with that of Firmicutes has been observed in most of the studies analyzed, and that increase in Actinobacteria was associated with better glycemic control or lipid profile at follow-up [187], hypothesizing the involvement of these two bacterial phyla on better glycemic control. In fact, the genus *Bifidobacterium*, belonging to the Actinobacteroidota phylum, has protective effects in T2DM as it is involved in the production of SCFA precursors and in the regulation of glucose homeostasis [189,190].

Concerning the comparative prediction analysis of the functional metagenome, in the KETO group, the strongest associations were related to steroid and carotenoid biosynthesis and non-homologous end joining (NHEJ) pathways both at T2 and T3 compared with baseline. Carotenoids are molecules with antioxidants and nutraceutical functions, beneficial for human health, to the point of considering the use of microbiological systems for their biotechnological production [191]. The NHEJ pathway constitutes a type of double-stranded DNA repair pathway (DSB) predominant in human cells that prevents genomic instability, and its deregulation can promote carcinogenesis [192]. Steroid hormones are used in the treatment and prophylaxis of various acute and chronic inflammatory and autoimmune diseases [193]. Despite their effectiveness, glucocorticoids (GC) have been associated with a high risk of developing hyperglycemia and overt DM [194,195,196]. In light of their side effects, further studies are needed to clarify the physiological significance of the increase in steroid biosynthesis in our patients, given that KETO showed, instead, an improvement in the anthropometric and metabolic status.

Overall, we have observed in KETO after NI the reduction in the degradation pathways of different alkylbenzenes, such as benzoate, ethylbenzene, and xylenes, while toluene was significantly increased at T2. Moreover, limonene and pinene degradation were strongly and negatively associated in the same cohort. The significance of the reduction in this pathway should be investigated to understand whether it can be considered a direct consequence of the diet or of the reduction in blood glucose levels. Indeed, limonene was shown to reduce hyperglycemia and attenuate diabetes-associated complications, while pinene has been shown to reduce hyperglycemia and hyperlipidemia and exert antioxidant activity in diabetic rats [197].

We also observed an increase in other glycan degradation pathways over time (significant at T2) and in two-component system pathways over time (significant at T3). A depletion in glycan metabolism in obese patients was observed in previous studies [32], [180,198], while two-component signal transduction systems, a communication system through which bacteria adapt their cellular physiology to changes in the environment, was found to be increased in healthy long-lived subjects [199].

Consistent with the results of the taxonomic analysis, no pathway was significantly associated with MEDI over time.

## 5. Conclusions

Our study suggested the potential benefits of a VLCKD protocol in drug-naïve patients with T2DM and overweight/obesity, at least in the short term. In particular, these benefits seem to be higher than those observed with a classical MD regarding weight loss and the impact on GM, although further investigations are needed.

Indeed, the VLCKD has shown greater improvements in anthropometric measures (weight, BMI, FM%, and WC) and in quality of life compared to the MD. Nevertheless, changes in metabolic variables were not statistically significant between the two diets. The results also highlighted an improvement in eating habits, with an increase in adherence to MD in both groups. The progressive shift to a Mediterranean-style diet after two months of KD allows patients not to be excessively restrictive about many food groups and to reduce their carbon footprint. Still, physical activity levels remained unchanged in both groups.

Consistently, our findings highlight a more beneficial impact of the VLCKD on the intestinal microbial phenotype, suggesting that this diet could be considered a valid approach to managing newly diagnosed diseases without drugs. Overall, in the KETO group, both after two months (ketosis phase) and after 3 months of NI (shift to MD), there was a significant increase in biomarkers of intestinal homeostasis, such as Verrucomicrobiota phylum with its members Verrucomicrobiae, Verrucomicrobiales, Akkermansiaceae, and *Akkermansia*, as well as in microbial taxa associated with a lean phenotype and with a healthy glucose metabolism, such as the Christensenellaceae family and in beneficial taxa capable of modulating gut inflammation through SCFAs production (*Eubacterium* spp.); while the reduction in microbial taxa previously associated with obesity (Firmicutes and Actinobacteriota) or other diseases (*Alistipes*) was observed. Greater clarity must be made regarding the association of the Peptococcaceae family due to the discordant data in the literature on their beneficial role.

The ketosis phase (T2) was associated with the reduction in taxa belonging to the Lachnospiraceae family, in taxa associated with GIT neoplasms and poor metabolic control (*Subdoligranulum*) and with the increase in taxa already shown to be enriched in diabetic mice undergoing treatment to alleviate type 2 diabetes through the modulation of intestinal microbiota. (*Intestinimonas*).

Phase T3 of the VLCKD was associated with taxa whose physiological role remains to be defined (UCG 010 family, unclassified *UCG 010* genus and species, and Clostridia_UCG-014), with taxa previously associated with physical activity in pathogen models (Oscillospiraceae UCG-005) and with the reduction in taxa for which a lower abundance in mice with T2DM following the anti-hyperglycemic treatments (*Lachnoclostridium*) and a lower abundance after bariatric surgery and diabetes remission (*Ruminococcus torques group*) had already been demonstrated.

An inconsistent trend (increase at T2 compared with baseline and decrease at T3 compared with T2, without variation between T0 and T3) was found for taxa associated with leanness and metabolic benefits (*Parabacteroides* and *P. distasonis*), although its administration has been shown to induce depressive-like behavior in mouse models.

On the other hand, our findings indicate that the MD, at least in the short term, has less impact on the GM of patients with T2DM and obesity compared to the VLCKD, the significance of which needs further investigation. We can hypothesize that for MD, a longer-lasting NI and greater weight loss are needed to induce more significant changes in GM composition.

Consistent with the results of the taxonomic analysis, no pathway was significantly associated with MEDI over time, whereas the strongest associations were related to steroid and carotenoid biosynthesis and to non-homologous end-joining pathways in KETO cohort; in the same cohort, penicillin and cephalosporin biosynthesis, limonene and pinene degradation and ethylbenzene degradation pathways were strongly and negatively associated.

Our study is limited by its small sample size due to the COVID-19 pandemic and especially to the lockdown period that forced the interruption of the enrollment of patients; moreover, a female patient assigned to the MEDI group has been excluded during the follow-up period due to the COVID-19 infection, further reducing the number of patients of MEDI group.

Therefore, despite the potential benefits obtained with the ketogenic diet in the short term, these findings need to be confirmed by larger study cohorts with longer follow-ups to validate the use of KD as an effective dietary model in the treatment of newly diagnosed T2DM with obesity. In the same way, greater sample sizes and longer-lasting therapeutic interventions are needed to confirm the impact of the Mediterranean diet in the same patient category and the extent of its effectiveness compared to the ketogenic diet.

## Figures and Tables

**Table 1:** Anthropometric, metabolic, lifestyle, and health status in KETO and MEDI groups at baseline.

Variable (M ± SD)	KETO	MEDI	*p*-Value
Body weight (kg)	95.2 ± 15.2	86.7 ± 16.8	0.4
BMI (kg/m^2^)	35.3 ± 4.3	30.2 ± 4.2	0.07
WC (cm)	115.8 ± 6.6	109 ± 8.6	0.2
FM (%)	38.5 ± 8.2	34.8 ± 8.6	0.5
FFM (kg)	59.1 ± 15.6	56.5 ± 12.3	0.8
phA (°)	6.1 ± 0.4	5.6 ± 0.5	0.07
FPG (mg/dL)	127.7 ± 29.4	133.8 ± 10.2	0.7
HbA1c (%)	6.6 ± 0.9	7.1 ± 0.9	0.4
Total Cholesterol (mg/dL)	215.5 ± 66.3	219.2 ± 11.6	0.9
LDL Cholesterol (mg/dL)	137.3 ± 57.0	139.8 ± 12.8	0.9
HDL Cholesterol (mg/dL)	48.7 ± 11.9	53.4 ± 9.2	0.5
Triglycerides (mg/dL)	146.3 ± 82.7	130.4 ± 73.1	0.7
SBP (mmHg)	133 ± 15.4	151.0 ± 11.4	0.06
DBP (mmHg)	78.5 ± 9.0	81.0 ± 10.8	0.7
MDS	25.7 ± 7.3	26.8 ± 4.6	0.8
PAL(METs/week)	915.8 ± 1190.8	708.0 ± 559.9	0.8
Daily sitting time (h/day)	6.0 ± 2.3	7.2 ±2.7	0.4
SF-36 PCS	40.8 ± 9.6	50.4 ± 4.2	0.07
SF-36 MCS	46.3 ± 11.9	49.0 ± 15.0	0.7

BMI = Body Mass Index; WC = Waist Circumference; FM = Fat Mass; FFM; Free Fat Mass; phA = phase Angle; FPG = fasting plasma glucose; HbA_1_c = glycosylated hemoglobin; SBP = Systolic Blood Pressure; DBP = Diastolic Blood Pressure; MDS = Mediterranean Diet Score; PAL = Physical Activity Level; METs/week = Metabolic Equivalent of Task-minutes per week; SF-36 PCS = Physical Component Summary of SF-36; SF-36 MCS = Mental Component Summary of SF-36.

**Table 2:** Anthropometric, metabolic, lifestyle, and health status evaluation in KETO and MEDI groups at T0 and T3.

Variable (M ± SD)	KETO			MEDI		
	T0	T3	*p*-Value	T0	T3	*p*-Value
Body weight (kg)	95.2 ± 15.1	80.9 ± 12.3	**<0.0001**	86.7 ± 16.8	83.6 ± 18.3	**0.02**
BMI (kg/m^2^)	35.3 ± 4.3	30.0 ± 3.4	**<0.0001**	30.2 ± 4.2	29.1 ± 4.8	**0.02**
WC (cm)	115.8 ± 6.6	103.5 ± 7.8	**<0.0002**	109 ± 8.6	104.3 ± 9.5	**0.004**
FM (%)	38.5 ± 8.2	31.5 ± 9.3	**0.001**	34.8 ± 8.6	31.8 ± 9.6	0.07
FFM (kg)	59.1 ± 15.6	56.2 ± 15.0	**0.01**	56.5 ± 12.3	56.7 ± 13.2	0.88
phA (°)	6.1 ± 0.4	5.9 ± 0.5	0.36	5.6 ± 0.5	5.9 ± 0.6	0.25
FPG (mg/dL)	127.7 ± 29.4	102.8 ± 17.5	0.08	133.8 ± 10.2	140.6 ± 23.5	0.57
HbA1c (%)	6.6 ± 0.9	5.5 ± 0.5	**0.012**	7.1 ± 0,9	6.4 ± 0.8	0.22
Total Cholesterol (mg/dL)	215.5 ± 66.3	168 ± 40.7	0.08	219.2 ± 11.6	210.2 ± 20.7	0.36
LDL Cholesterol (mg/dL)	137.3 ± 57.0	103.6 ± 39.1	0.13	139.8 ± 12.8	124.2 ± 12.4	0.09
HDL Cholesterol (mg/dL)	48.7 ± 11.9	48.8 ± 9.1	0.9	53.4 ± 9.2	59.6 ± 8.4	0.16
Triglycerides (mg/dL)	146.3 ± 82.7	62.5 ± 19.4	0.08	130.4 ± 73.1	131.6 ± 96.4	0.92
SBP (mmHg)	133.0 ± 15.4	139.0 ± 15.9	0.14	151.0 ± 11.4	147.0 ± 17.2	0.33
DBP (mmHg)	78.5 ± 9.0	80.8 ± 3.8	0.45	81.0 ± 10.8	85.0 ± 13.5	0.5
MDS	25.7 ± 7.3	30 ± 5.7	0.2	26.8 ± 4.6	30.4 ± 5.6	0.4
PAL (METs/week)	915.8 ± 1190.8	755 ± 590.4	0.78	708.0 ± 559.9	754 ± 501	0.92
Daily sitting time (h/day)	6 ± 2.3	5.7 ± 1.9	0.57	7.2 ± 2.7	6.6 ± 3.3	0.6
SF-36 PCS	40.8 ± 9.6	46.8 ± 8.8	**0.05**	50.4 ± 4.2	46.6 ± 8.1	0.25
SF-36 MCS	46.3 ± 11.9	53.9 ± 8.8	**0.03**	49.0 ± 15.0	40.8 ± 9.6	0.23

Bold values denote statistical significance at the *p* < 0.05 level. BMI = Body Mass Index; WC = Waist Circumference; FM = Fat Mass; FFM; Free Fat Mass; phA = phase Angle; FPG = fasting plasma glucose; HbA_1_c = glycosylated hemoglobin; SBP = Systolic Blood Pressure; DBP = Diastolic Blood Pressure; MDS = Mediterranean Diet Score; PAL = Physical Activity Level; METs/week = Metabolic Equivalent of Task-minutes per week; SF-36 PCS = Physical Component Summary of SF-36; SF-36 MCS = Mental Component Summary of SF-36.

**Table 3:** Anthropometric, metabolic, lifestyle, and health status T0–T3 variations in KETO and MEDI groups.

Variable (Δ T0–T3)	KETO	MEDI	*p*-Value
Body weight (kg)	−14.3 ± 3.1	−3.04 ± 1.9	**<0.0001**
BMI (kg/m^2^)	−5.3 ± 0.9	−1.1 ± 0.7	**<0.0001**
WC (cm)	−12.9 ± 3.1	−4.7 ± 1.8	**0.0006**
FM (%)	−7 ± 2.7	−3.1 ± 2.7	**0.03**
FFM (kg)	−2.8 ± 1.8	+0. 2 ± 2.8	0,053
phA (°)	−0.2 ± 0.2	+0.3 ± 0.5	**0.04**
FPG (mg/dL)	−24.8 ± 27.9	+6.8 ± 25.3	0.08
HbA1c (%)	−1.15 ± 0.7	−0.7 ± 1.1	0.45
Total Cholesterol (mg/dL)	−47.5 ± 54.9	−3.8 ± 21.7	0.13
LDL Cholesterol (mg/dL)	−30.5 ± 47.2	−15.6 ± 16.3	0.52
HDL Cholesterol (mg/dL)	+0.17 ± 6.1	+6.2 ± 8.1	0.19
Triglycerides (mg/dL)	−83.8 ± 96.4	−1.2 ± 27	0.09
SBP (mmHg)	+6.5 ± 9.3	−4.0 ± 8.2	0.08
DBP (mmHg)	+4.0 ± 7.6	+4.0 ± 13.9	0.99
MDS	+6 ± 5.9	+3.6 ± 8.6	0.59
PAL (METs/week)	+19.2 ± 1263	+46 ± 1001	0.97
Daily sitting time (h/day)	−0.3 ±1.4	−0.6 ± 2.4	0.8
SF-36 PCS	+6.2 ± 3.1	−3.8 ± 6.4	**0.007**
SF-36 MCS	+7.5 ± 6.8	−8.2 ± 13.3	**0.03**

Bold values denote statistical significance at the *p* < 0.05 level. BMI = Body Mass Index; WC = Waist Circumference; FM = Fat Mass; FFM; Free Fat Mass; phA = phase Angle; FPG = fasting plasma glucose; HbA_1_c = glycosylated hemoglobin; SBP = Systolic Blood Pressure; DBP = Diastolic Blood Pressure; MDS = Mediterranean Diet Score; PAL = Physical Activity Level; METs/week = Metabolic Equivalent of Task-minutes per week; SF-36 PCS = Physical Component Summary of SF-36; SF-36 MCS = Mental Component Summary of SF-36.

**Table 4:** Changes in gut microbiota taxa abundances between timepoints in the KETO group.

Phylum	Class	Order	Family	Genus	Species	Ref. Group	*p*	*q*	↓/↑	Coeff.	Std.Err.
Actinobacteriota						T0–T2	0.073	0.218	↓	−1.85	0.92
Actinobacteriota						T0–T3	0.087	0.224	↓	−1.75	0.92
Bacteroidota						T0–T3	0.068	0.218	↓	−0.64	0.32
	Bacteroidia	Bacteroidales				T0–T3	0.048	0.242	↓	−0.70	0.31
			Tannerellaceae			T0–T2	0.016	0.136	↑	1.71	0.59
			Tannerellaceae			T2–T3	0.029	0.179	↓	−1.50	0.59
				Parabacteroides		T0–T2	0.043	0.220	↑	1.40	0.60
				Parabacteroides		T2–T3	0.027	0.204	↓	−1.57	0.60
					Parabacteroides_distasonis	T0–T2	0.007	0.113	↑	2.36	0.69
					Parabacteroides_distasonis	T2–T3	0.027	0.226	↓	−1.79	0.69
			Bacteroidaceae	Bacteroides		T0–T3	0.018	0.203	↓	−1.01	0.36
			Barnesiellaceae	Barnesiella		T0–T3	0.034	0.204	↓	−2.44	1.00
			Rikenellaceae	Alistipes		T0–T2	0.011	0.203	↓	−1.31	0.42
			Rikenellaceae	Alistipes		T0–T3	0.032	0.204	↓	−1.05	0.42
					Alistipes_shahii	T0–T2	0.023	0.199	↓	−1.66	0.62
			Marinifilaceae	Butyricimonas		T0–T3	0.041	0.220	↓	−1.51	0.64
Firmicutes						T0–T2	0.009	0.120	↓	−0.74	0.23
Firmicutes						T0–T3	0.050	0.218	↓	−0.51	0.23
	Clostridia					T0–T2	0.015	0.162	↓	−0.55	0.19
		Peptostreptococcales.Tissierellales				T0–T2	0.013	0.129	↓	−1.42	0.47
		Christensenellales				T0–T2	0.010	0.129	↑	4.00	1.25
		Christensenellales				T0–T3	0.008	0.129	↑	4.14	1.25
			Christensenellaceae			T0–T2	0.010	0.131	↑	3.99	1.25
			Christensenellaceae			T0–T3	0.008	0.131	↑	4.16	1.25
				Christensenellaceae_R.7_group		T0–T2	0.014	0.203	↑	2.16	0.72
				Christensenellaceae_R.7_group		T0–T3	0.011	0.203	↑	2.24	0.72
					Genus_Christensenellaceae_R.7_group	T0–T2	0.005	0.110	↑	2.57	0.72
					Genus_Christensenellaceae_R.7_group	T0–T3	0.004	0.110	↑	2.70	0.72
		Clostridia_UCG.014				T0–T3	0.013	0.129	↑	1.87	0.62
			Clostridia_UCG.014			T0–T3	0.012	0.136	↑	1.89	0.62
				Clostridia_UCG.014		T0–T3	0.024	0.204	↑	1.70	0.64
					Genus_Clostridia_UCG.014	T0–T3	0.009	0.113	↑	2.21	0.68
		Oscillospirales				T0–T2	0.019	0.135	↓	−0.85	0.30
			Ruminococcaceae			T0–T2	0.008	0.131	↓	−1.30	0.39
				Subdoligranulum		T0–T2	0.019	0.203	↓	−1.88	0.67
			UCG.010			T0–T3	0.009	0.131	↑	1.26	0.39
			UCG.010			T2–T3	0.002	0.131	↑	1.60	0.39
				UCG.010		T0–T3	0.048	0.226	↑	1.05	0.46
				UCG.010		T2–T3	0.007	0.203	↑	1.57	0.46
					Genus_UCG.010	T0–T3	0.002	0.110	↑	1.62	0.39
					Genus_UCG.010	T2–T3	0.003	0.110	↑	1.55	0.39
			Oscillospiraceae	Intestinimonas		T0–T2	0.025	0.204	↑	2.69	1.02
				UCG.005	Genus_UCG.005	T0–T3	0.016	0.157	↑	2.54	0.87
			Lachnospiraceae	X.Eubacterium._xylanophilum_group		T0–T2	0.046	0.226	↑	1.49	0.65
			Lachnospiraceae	X.Eubacterium._xylanophilum_group		T0–T3	0.002	0.203	↑	2.69	0.65
					Genus_.Eubacterium._xylanophilum_group	T0–T2	0.009	0.113	↑	1.81	0.56
			Lachnospiraceae		Genus_.Eubacterium._xylanophilum_group	T0–T3	0.000	0.039	↑	3.06	0.56
			Lachnospiraceae	X.Eubacterium._eligens_group		T0–T2	0.018	0.203	↑	2.78	1.05
			Lachnospiraceae	X.Eubacterium._eligens_group		T0–T3	0.032	0.204	↑	2.49	1.05
			Lachnospiraceae		Genus_.Eubacterium._eligens_group	T0–T2	0.005	0.110	↑	3.28	0.99
					Genus_.Eubacterium._eligens_group	T0–T3	0.008	0.113	↑	3.03	0.99
			Lachnospiraceae	Lachnoclostridium		T0–T3	0.042	0.220	↓	−1.74	0.75
			Lachnospiraceae	Lachnoclostridium		T2–T3	0.006	0.203	↓	−2.57	0.75
					Genus_Lachnoclostridium	T2–T3	0.009	0.113	↓	−2.48	0.77
			Lachnospiraceae	Anaerostipes		T0–T2	0.033	0.204	↓	−2.37	0.96
			Lachnospiraceae	Dorea		T0–T2	0.032	0.204	↓	−1.85	0.74
			Lachnospiraceae	Agathobacter		T0–T2	0.013	0.203	↓	−2.65	0.87
			Lachnospiraceae	Fusicatenibacter		T0–T2	0.016	0.203	↓	−2.12	0.73
			Lachnospiraceae	X.Ruminococcus._torques_group		T0–T3	0.041	0.220	↓	−1.40	0.60
		Peptococcales				T0–T2	0.026	0.143	↑	2.24	0.85
			Peptococcaceae			T0–T2	0.025	0.175	↑	2.24	0.85
				Family_Peptococcaceae		T0–T2	0.033	0.204	↑	2.06	0.83
					Family_Peptococcaceae	T0–T2	0.011	0.123	↑	2.73	0.88
					Family_Peptococcaceae	T0–T3	0.021	0.198	↑	2.41	0.88
Verrucomicrobiota						T0–T2	0.014	0.120	↑	5.07	1.70
Verrucomicrobiota						T0–T3	0.020	0.120	↑	4.69	1.70
	Verrucomicrobiae					T0–T2	0.014	0.162	↑	5.06	1.70
	Verrucomicrobiae					T0–T3	0.020	0.162	↑	4.69	1.70
		Verrucomicrobiales				T0–T2	0.014	0.129	↑	5.03	1.70
		Verrucomicrobiales				T0–T3	0.021	0.135	↑	4.65	1.70
			Akkermansiaceae			T0–T2	0.014	0.136	↑	5.02	1.69
			Akkermansiaceae			T0–T3	0.020	0.157	↑	4.66	1.69
				Akkermansia		T0–T2	0.016	0.203	↑	4.72	1.63
				Akkermansia		T0–T3	0.024	0.204	↑	4.34	1.63

Statistical significance was evaluated by running a Generalized Linear Mixed-effects Model with MaAsLin2. Effect size is represented by the MaAsLin2 model coefficients and respective standard errors. Only taxa abundance changes at *p* ≤ 0.05 and *q* ≤ 0.25 are considered statistically significant. *q*: *p* adjusted for Benjamini–Hochberg (BH) correction test with a cut-off at *q* ≤ 0.25. Samples were analyzed at baseline (T0), after two months (T2), and after three months (T3) of the nutritional intervention. KETO = 6 patients who followed a very-low-calorie ketogenic diet (VLCKD); Ref. group = time points compared; ↓ = significantly reduced in the second term of the pairwise group; ↑ = significantly increased in the second term of the pairwise group.

**Table 5:** Changes in gut microbiota taxa abundances between timepoints in the MEDI group.

Phylum	Class	Order	Family	Genus	Species	Ref. Group	*p*	*q*	↓/↑	Coeff.	Std._Err.
Actinobacteriota						T0-T3	0.029	0.212	↑	1.90	0.71
Actinobacteriota						T2-T3	0.056	0.212	↑	1.59	0.71
Firmicutes						T2-T3	0.057	0.212	↑	0.61	0.27
Desulfobacterota						T2-T3	0.048	0.212	↓	−2.45	1.05
Bacteroidota	Bacteroidia	Bacteroidales	Bacteroidaceae	Bacteroides	Genus_Bacteroides	T2-T3	0.002	0.246	↓	−2.17	0.49

Statistical significance was evaluated by running a Generalized Linear Mixed-effects Model with MaAsLin2. Effect size is represented by the MaAsLin2 model coefficients and respective standard errors. Only taxa abundance changes at *p* ≤ 0.05 and *q* ≤ 0.25 are considered statistically significant. *q*: *p* adjusted for Benjamini–Hochberg (BH) correction test with a cut-off at *q* ≤ 0.25. Samples were analyzed at baseline (T0), after two months (T2), and after three months (T3) of nutritional intervention. MEDI = 5 patients who followed a low-calorie Mediterranean diet (MD); Ref. group = time points compared; ↓ = significantly reduced in the second term of the pairwise group; ↑ = significantly increased in the second term of the pairwise group.

## Data Availability

Our sequence data for the 16S rRNA gene was deposited in the European Nucleotide Archive (ENA) (https://www.ebi.ac.uk/ena, accessed on 29 September 2022), under the study accession number PRJEB56373 (ERP141305); https://www.ebi.ac.uk/ena/browser/view/PRJEB56373 (accessed on 29 September 2022).

## Supplementary Materials

Supporting information can be downloaded at: https://www.mdpi.com/article/10.3390/metabo12111092/s1. Nutritional evaluation; Table S1: Nutritional analysis in Keto and Medi Groups; Table S2: Alpha diversity analysis between KETO and MEDI; Table S3: Alpha diversity analysis in KETO over time; Table S4: Alpha diversity analysis in MEDI over time; Table S5: GM beta diversity analysis between KETO and MEDI; Table S6: GM beta diversity analysis in KETO over time; Table S7: GM beta diversity analysis in MEDI over time; Table S8: Firmicutes/Bacteroidota ratio analysis in KETO over time; Table S9: Firmicutes/Bacteroidota ratio analysis in MEDI over time; Table S10: Changes in gut microbiota predicted function abundances between timepoints in KETO group.
