# Supplementary material for: Dynamics of Gut Microbiota and Clinical Variables after Ketogenic and Mediterranean Diets in Drug-Naïve Patients with Type 2 Diabetes Mellitus and Obesity
Source: Metabolites. 2022 Nov 10;12(11):1092. doi: 10.3390/metabo12111092 (PMC9693465; doi:10.3390/metabo12111092)
Supplement: Supplementary file 1 [file metabolites-12-01092-s001.zip › metabolites-1983709-supplementary.pdf]

Supplementary Materials

Dynamics of Gut Microbiota and Clinical Variables after Ketogenic and Mediterranean Diets in Drug-Naïve Patients with Type 2 Diabetes Mellitus and Obesity

Materials and Methods

Nutritional evaluation

The Med Diet Score, considers 11 food groups, and assigns an increasing score from 0 to 5, on the basis of the monthly or weekly consumption of foods considered typical of the Mediterranean pattern (unrefined cereals, potatoes, vegetables, fruit, legumes, fish and olive oil), and a decreasing score from 5 to 0, on the basis of the monthly consumption of foods considered away from the same model (meat or derivatives, poultry, whole dairy products); as for alcohol consumption, decreasing scores from 5 to 1 correspond respectively to a consumption <300 ml/day, <400 ml/day, <500 ml/day, <600 ml/day, while a score of 0 corresponds to a consumption > 700 ml/d or no consumption. The total score, obtained from the sum of the 11 individual scores (one for each food group), ranges from 0 to 55, and higher values indicate a higher adherence to MD.

The analysis of the food diary allowed us to estimate the following parameters: average daily caloric intake expressed in Kilocalories (Kcal), daily percentage of carbohydrates and lipids intake, daily intake of proteins in grams, and daily intake of fibers in grams. In addition, the food diary was also useful for obtaining information regarding the "eating style" of the patients (number of meals during the day, intervals between them, place where meals are consumed, predilection of certain foods, possible nocturnal or compulsive nutrition).

Table S1. Nutritional analysis in Keto and Medi Groups.

Dietary parameters (M± SD)	KETO GROUP			MEDI GROUP		
	T0	T3	*p*-value	T0	T3	*p*-value
Food intake Kcal/day	1516 ± 367	1161± 206	0.09	1840 ± 281	1630 ± 104	0.2
Carbohydrates %	42.5 ± 3.1	34.7± 5.2	0.0501	42.0 ± 8.6	45.8 ± 4.1	0.5
Proteins %	19.7 ± 2.1	26.7 ± 2.6	0.004	19.6 ± 2.9	19.0 ± 2.6	0.6
Lipids %	38.8 ± 2.9	40 ± 4.3	0.6	37.8 ± 8.1	35.2 ± 2.3	0.6
Fibres g	13.8 ± 6.7	15.5 ± 5.0	0.4	20.8 ± 5.8	22.8 ±7.6	0.5
Non refined cereals	1.8 ± 2.5	4.2 ± 1.0	0.1	1.4 ± 1.7	2.6 ± 2.1	0.2
Potatoes	1.2 ± 0.7	0.3 ± 0.8	0.04	1.0 ± 0	2.0 ± 1.7	0.2
Vegetables	3.3 ± 1.5	5 ± 0	0.04	4.2 ± 1.8	5.0 ± 0	0.4
Fruit	3.8 ± 1.9	4.0 ± 1.7	0.9	3.8 ± 2.2	3.8 ± 1.8	1.0
Legumes	1.3 ± 0.5	1.7 ± 1.0	0,5	1.2 ± 0.5	1.6 ± 1.1	0.5
Fish	1.7 ± 0.8	2.8 ± 0.7	0.01	2.1 ± 0.9	2.4 ± 0.9	1.0
Red Meat	2.0 ± 1.7	2.5 ± 1.4	0.4	1.8 ± 0.8	2.4 ± 1.1	0.2
Poultry	2.3 ± 1.4	1.5 ± 0.6	0.2	2.8 ± 1.6	3.0 ± 1.4	0.8
Full fat dairy	1.8 ± 1.5	2.0 ± 1.7	0.7	2.0 ± 1.6	1.6 ± 1.1	0.7
Extra virgin olive oil	4.7 ± 0.8	5.0 ± 0	0.4	5.0 ± 0	5.0 ± 0	1.0
Alcohol	1.7 ± 2.6	0.8 ± 2.0	0.6	1.6 ± 2.3	1.0 ± 2.2	0.6
MDS	25.7 ± 7.3	30.0 ± 5.7	0.2	26.8 ± 4.6	30.4 ± 5.6	0.4

Food Intake (kcal/die), macronutrients composition (%), fibres intake (g) are derived from the three day food diary; the intake of each of the 11 food groups included in the Med Diet Score, is expressed as a score 0–5; MDS is the total score ranging 0–55, statistical analysis: paired t test; p value (p) < 0.05 is considered statistically significant

Table S2. Alpha diversity analysis between KETO and MEDI

**Shannon**	*p*
**T0 (KETO vs MEDI)**	0.052
**T2 (KETO vs MEDI)**	0.662
**T3 (KETO vs MEDI)**	0.792
**Observed ASVs**	*p*
**T0 (KETO vs MEDI)**	0.931
**T2 (KETO vs MEDI)**	0.410
**T3 (KETO vs MEDI)**	0.537
**Evenness (Pielou's J)**	*p*
**T0 (KETO vs MEDI)**	0.017
**T2 (KETO vs MEDI)**	0.792
**T3 (KETO vs MEDI)**	0.792

Differences in the Shannon index (H'), Observed ASVs and evenness (Pielou's index: J') between KETO and MEDI were assessed by Mann-Whitney U test using the stats R package. p equal to or less than 0.05 was considered statistically significant. KETO= 6 patients who followed a very low calorie ketogenic diet, MEDI=5 patients who followed a low-calorie Mediterranean diet. IQR, interquartile range.

Table S3. Alpha diversity analysis in KETO over time

**Shannon**	**Median (IQR)**	***p* (*vs* T2)**	***p* (*vs* T3)**
**T0**	3.77 (0.17)	0.688	0.563
**T2**	3.57 (0.63)		0.438
**T3**	3.30 (0.71)		
**Observed ASVs**	**Median (IQR)**	***p* (*vs* T2)**	***p* (*vs* T3)**
**T0**	171.5 (23.75)	0.293	0.688
**T2**	200.5 (22.25)		0.219
**T3**	190 (22.25)		
**Evenness (Pielou's J)**	**Median (IQR)**	***p* (*vs* T2)**	***p* (*vs* T3)**
**T0**	0.74 (0.06)	0.438	0.438
**T2**	0.68 (0.12)		0.563
**T3**	0.64 (0.15)		

Differences in the Shannon index (H'), Observed ASVs and evenness (Pielou's index: J') in KETO before (T0), after two months (T2) and after three months (T3) of very low calorie ketogenic diet were assessed by the paired Wilcoxon signed-rank test using the *stats* R package. *p* equal to or less than 0.05 was considered statistically significant. KETO= 6 patients who followed a very low calorie ketogenic diet. IQR, interquartile range.

Table S4. Alpha diversity analysis in MEDI over time

**Shannon**	**Median (IQR)**	***p* (*vs* T2)**	***p* (*vs* T3)**
**T0**	3.08 (0.42)	0.313	1
**T2**	3.16 (0.70)		0.625
**T3**	3.38 (1.09)		
**Observed ASVs**	**Median (IQR)**	***p* (*vs* T2)**	***p* (*vs* T3)**
**T0**	172 (35)	0.625	1
**T2**	194 (24)		1
**T3**	180 (9)		
**Evenness (Pielou's J)**	**Median (IQR)**	***p* (*vs* T2)**	***p* (*vs* T3)**
**T0**	0.61 (0.06)	0.188	1
**T2**	0.61 (0.10)		0.625
**T3**	0.64 (0.20)		

Differences in the Shannon index (H’), richness (S) and evenness (Pielou’s index: J’) in MEDI before (T0), after two months (T2) and after three months (T3) of low-calorie Mediterranean diet were assessed by by the paired Wilcoxon signed-rank test using the *stats* R package. *p* equal to or less than 0.05 was considered statistically significant. MEDI= 5 patients who followed a low-calorie Mediterranean diet. IQR, interquartile range.

Table S5. GM beta diversity analysis between KETO and MEDI

**Bray-Curtis**	**T0 (KETO *vs* MEDI)**	**T2 (KETO *vs* MEDI)**	**T3 (KETO *vs* MEDI)**
**Degrees of freedom**	1	1	1
**Sum of squares**	0.599	0.308	0.316
**Mean of squares**	0.599	0.308	0.316
**F**	1.727	0.830	0.848
R^2	0.161	0.084	0.086
p	0.013	0.696	0.656
**Unweighted UniFrac**	**T0 (KETO *vs* MEDI)**	**T2 (KETO *vs* MEDI)**	**T3 (KETO *vs* MEDI)**
**Degrees of freedom**	1	1	1
**Sum of squares**	0.118	0.125	0.144
**Mean of squares**	0.118	0.125	0.144
**F**	0.838	0.869	1.079
R^2	0.085	0.088	0.107
p	0.864	0.753	0.357
**Weighted UniFrac**	**T0 (KETO *vs* MEDI)**	**T2 (KETO *vs* MEDI)**	**T3 (KETO *vs* MEDI)**
**Degrees of freedom**	1	1	1
**Sum of squares**	0.163	0.063	0.047
**Mean of squares**	0.163	0.063	0.047
**F**	1.724	0.480	0.490
R^2	0.161	0.051	0.052
p	0.150	0.674	0.695

Differences in the beta diversity index between KETO and MEDI were evaluated by Permutational Multivariate Analysis of Variance (PERMANOVA), performed using the *vegan* R package. Samples were analyzed at baseline (T0), after two months (T2) and after three months (T3) of the of nutritional intervention. p equal to or less than 0.05 was considered statistically significant. KETO= 6 patients who followed a very low calorie ketogenic diet, MEDI= 5 patients who followed a low-calorie Mediterranean diet.

Table S6. GM beta diversity analysis in KETO over time

**Bray-Curtis**	**T0 *vs* T2**	**T0 *vs* T3**	**T2 *vs* T3**
**Degrees of freedom**	1	1	1
**Sum of squares**	0.318	0.319	0.080
**Mean of squares**	0.318	0.319	0.080
**F**	0.830	0.832	0.206
**R^2**	0.077	0.077	0.020
p	0.737	0.711	0.958
**Unweighted UniFrac**	**T0 *vs* T2**	**T0 *vs* T3**	**T2 *vs* T3**
**Degrees of freedom**	1	1	1
**Sum of squares**	0.116	0.104	0.057
**Mean of squares**	0.116	0.104	0.057
**F**	0.802	0.758	0.423
**R^2**	0.074	0.070	0.041
p	0.733	0.828	0.934
**Weighted UniFrac**	**T0 *vs* T2**	**T0 *vs* T3**	**T2 *vs* T3**
**Degrees of freedom**	1	1	1
**Sum of squares**	0.166	0.196	0.019
**Mean of squares**	0.166	0.196	0.019
**F**	1.960	2.657	0.208
**R^2**	0.164	0.210	0.020
p	0.121	0.067	0.844

Differences in the beta diversity index in KETO before (T0), after two months (T2) and after three months (T3) of very low calorie ketogenic diet were evaluated by Permutational Multivariate Analysis of Variance (PERMANOVA), performed using the *vegan* R package. *p* equal to or less than 0.05 was considered statistically significant. KETO= 6 patients who followed a very low calorie ketogenic diet.

Table S7. GM beta diversity analysis in MEDI over time

**Bray-Curtis**	**T0 *vs* T2**	**T0 *vs* T3**	**T2 *vs* T3**
**Degrees of freedom**	1	1	1
**Sum of squares**	0.099	0.117	0.107
**Mean of squares**	0.099	0.117	0.107
**F**	0.299	0.356	0.305
**R^2**	0.036	0.043	0.037
p	0.927	0.972	0.950
**Unweighted UniFrac**	**T0 *vs* T2**	**T0 *vs* T3**	**T2 *vs* T3**
**Degrees of freedom**	1	1	1
**Sum of squares**	0.048	0.058	0.040
**Mean of squares**	0.048	0.058	0.040
**F**	0.344	0.423	0.284
**R^2**	0.041	0.050	0.035
p	0.949	0.948	1
**Weighted UniFrac**	**T0 *vs* T2**	**T0 *vs* T3**	**T2 *vs* T3**
**Degrees of freedom**	1	1	1
**Sum of squares**	0.039	0.029	0.087
**Mean of squares**	0.039	0.029	0.087
**F**	0.260	0.235	0.622
**R^2**	0.031	0.028	0.072
p	0.768	0.939	0.657

Differences in the beta diversity index in MEDI before (T0), after two months (T2) and after three months (T3) of low-calorie Mediterranean diet were evaluated by Permutational Multivariate Analysis of Variance (PERMANOVA), performed using the *vegan* R package. *p* equal to or less than 0.05 was considered statistically significant. MEDI= 5 patients who followed a low-calorie Mediterranean diet.

Table S8. Firmicutes/Bacteroidota ratio analysis in KETO over time

Firmicutes/Bacteroidota	Median (IQR)	*p* (*vs* T2)	*p* (*vs* T3)
**T0**	1.56 (1.60)	0.844	1
**T2**	1.42 (1.19)		0.563
**T3**	1.97 (0.77)		

Differences in the Firmicutes/Bacteroidota ratio in KETO before (T0), after two months (T2) and after three months (T3) of very low calorie ketogenic diet evaluated by the paired Wilcoxon signed-rank test using the *stats* R package. *p* equal to or less than 0.05 was considered statistically significant. KETO= 6 patients who followed a very low calorie ketogenic diet. IQR, interquartile range.

Table S9. Firmicutes/Bacteroidota ratio analysis in MEDI over time

Firmicutes/Bacteroidota	Median (IQR)	*p* (*vs* T2)	*p* (*vs* T3)
**T0**	1.56 (1.27)	0.625	0.625
**T2**	1.06 (0.90)		0.625
**T3**	2.97 (0.58)		

Differences in the Firmicutes/Bacteroidota ratio in MEDI before (T0), after two months (T2) and after three months (T3) of very low calorie ketogenic diet evaluated by the paired Wilcoxon signed-rank test using the *stats* R package. *p* equal to or less than 0.05 was considered statistically significant. MEDI= 5 patients who followed a very low calorie ketogenic diet. IQR, interquartile range.

Table S10. Changes in gut microbiota predicted function abundances between timepoints in KETO group.

Pathway	Diet	Group 1 (reference)	Group 2	*p*	*q*	↓/↑	MaAsLin2 coeff.	Std err.
Methane.metabolism	KETO	T0	T3	<0.001	0.002	↓	-0.25	0.03
Methane.metabolism	KETO	T0	T2	<0.001	0.006	↓	-0.21	0.03
Cell.motility.and.secretion	KETO	T0	T2	<0.001	0.028	↑	0.49	0.09
Cytoskeleton.proteins	KETO	T0	T2	<0.001	0.028	↓	-0.23	0.04
Energy.metabolism	KETO	T0	T3	<0.001	0.028	↓	-0.16	0.03
Peptidases	KETO	T0	T3	<0.001	0.028	↓	-0.15	0.03
Protein.folding.and.associated.processing	KETO	T0	T2	<0.001	0.028	↑	0.16	0.03
Peptidases	KETO	T0	T2	<0.001	0.037	↓	-0.13	0.03
Cell.motility.and.secretion	KETO	T0	T3	<0.001	0.040	↑	0.45	0.09
Carotenoid.biosynthesis	KETO	T0	T2	0.001	0.051	↑	3.68	0.84
Cytoskeleton.proteins	KETO	T0	T3	0.001	0.051	↓	-0.19	0.04
Fructose.and.mannose.metabolism	KETO	T0	T2	0.001	0.051	↑	0.13	0.03
Limonene.and.pinene.degradation	KETO	T0	T2	0.001	0.051	↓	-0.77	0.16
Lipoic.acid.metabolism	KETO	T0	T2	0.001	0.051	↑	0.73	0.17
Lysine.degradation	KETO	T0	T2	0.001	0.051	↑	0.34	0.07

Lysine.degradation	KETO	T0	T3	0.001	0.051	↑	0.33	0.07
Non.homologous.end.joining	KETO	T0	T2	0.001	0.051	↑	2.34	0.54
Penicillin.and.cephalosporin.biosynthesis	KETO	T0	T2	0.001	0.051	↓	-1.29	0.30
Protein.folding.and.associated.processing	KETO	T0	T3	0.001	0.051	↑	0.13	0.03
Valine..leucine.and.isoleucine.degradation	KETO	T0	T2	0.001	0.051	↑	0.34	0.07
Limonene.and.pinene.degradation	KETO	T0	T3	0.002	0.053	↓	-0.71	0.16
Membrane.and.intracellular.structural.molecules	KETO	T0	T2	0.002	0.072	↑	0.25	0.06
Transcription.factors	KETO	T0	T2	0.002	0.072	↓	-0.20	0.05
Bacterial.motility.proteins	KETO	T0	T3	0.003	0.074	↑	0.71	0.20
Biosynthesis.of.siderophore.group.nonribosomal.peptides	KETO	T0	T2	0.004	0.095	↑	0.98	0.26
Chromosome	KETO	T0	T3	0.003	0.095	↓	-0.09	0.02
Citrate.cycle..TCA.cycle.	KETO	T0	T2	0.004	0.095	↑	0.18	0.05
Non.homologous.end.joining	KETO	T0	T3	0.004	0.095	↑	2.01	0.54
Riboflavin.metabolism	KETO	T0	T2	0.004	0.095	↑	0.21	0.06
Steroid.biosynthesis	KETO	T0	T2	0.004	0.095	↑	6.14	1.66
Valine..leucine.and.isoleucine.degradation	KETO	T0	T3	0.004	0.095	↑	0.27	0.07
Carbon.fixation.in.photosynthetic.organisms	KETO	T0	T3	0.005	0.097	↓	-0.07	0.02
Insulin.signaling.pathway	KETO	T0	T3	0.004	0.097	↑	0.40	0.12
Riboflavin.metabolism	KETO	T0	T3	0.005	0.097	↑	0.20	0.06
RNA.degradation	KETO	T0	T3	0.005	0.097	↑	0.14	0.04
Translation.factors	KETO	T0	T2	0.005	0.097	↓	-0.07	0.02
Restriction.enzyme	KETO	T0	T3	0.005	0.100	↓	-0.40	0.11
Amino.acid.metabolism	KETO	T0	T2	0.007	0.104	↑	0.36	0.11
Carotenoid.biosynthesis	KETO	T0	T3	0.006	0.104	↑	2.88	0.84
Energy.metabolism	KETO	T0	T2	0.007	0.104	↓	-0.11	0.03
Fructose.and.mannose.metabolism	KETO	T0	T3	0.006	0.104	↑	0.09	0.03
Lipoic.acid.metabolism	KETO	T0	T3	0.006	0.104	↑	0.58	0.17
Naphthalene.degradation	KETO	T0	T2	0.006	0.104	↓	-0.31	0.09
Penicillin.and.cephalosporin.biosynthesis	KETO	T0	T3	0.006	0.104	↓	-1.04	0.30
Phenylalanine.metabolism	KETO	T0	T3	0.007	0.104	↓	-0.20	0.06
RNA.degradation	KETO	T0	T2	0.006	0.104	↑	0.13	0.04
Histidine.metabolism	KETO	T0	T3	0.007	0.112	↓	-0.14	0.04

Base.excision.repair	KETO	T0	T3	0.008	0.113	↑	0.05	0.02
Biosynthesis.of.vancomycin.group.antibiotics	KETO	T0	T3	0.008	0.113	↑	0.20	0.06
Naphthalene.degradation	KETO	T0	T3	0.008	0.113	↓	-0.30	0.09
Photosynthesis.proteins	KETO	T0	T3	0.008	0.113	↑	0.28	0.08
Translation.factors	KETO	T0	T3	0.008	0.113	↓	-0.06	0.02
Chromosome	KETO	T0	T2	0.008	0.113	↓	-0.08	0.02
Bisphenol.degradation	KETO	T0	T2	0.011	0.131	↑	0.32	0.10
Histidine.metabolism	KETO	T0	T2	0.011	0.131	↓	-0.13	0.04
Insulin.signaling.pathway	KETO	T0	T2	0.011	0.131	↑	0.35	0.12
Linoleic.acid.metabolism	KETO	T0	T2	0.011	0.131	↑	0.32	0.10
Lysosome	KETO	T0	T2	0.012	0.131	↑	0.93	0.30
Photosynthesis	KETO	T0	T3	0.011	0.131	↑	0.27	0.09
Porphyrin.and.chlorophyll.metabolism	KETO	T0	T2	0.011	0.131	↑	0.25	0.08
Porphyrin.and.chlorophyll.metabolism	KETO	T0	T3	0.012	0.131	↑	0.25	0.08
Restriction.enzyme	KETO	T0	T2	0.011	0.131	↓	-0.35	0.11
Secretion.system	KETO	T0	T3	0.010	0.131	↑	0.11	0.03
Base.excision.repair	KETO	T0	T2	0.012	0.132	↑	0.05	0.02
Biosynthesis.of.siderophore.group.nonribosomal.peptides	KETO	T0	T3	0.013	0.132	↑	0.79	0.26
Carbohydrate.digestion.and.absorption	KETO	T0	T3	0.013	0.132	↓	-1.57	0.52
Ethylbenzene.degradation	KETO	T0	T3	0.012	0.132	↓	-0.84	0.27
Prenyltransferases	KETO	T0	T2	0.012	0.132	↑	0.18	0.06
Steroid.biosynthesis	KETO	T0	T3	0.014	0.141	↑	4.95	1.66
General.function.prediction.only	KETO	T0	T3	0.015	0.156	↓	-0.05	0.02
Glycosaminoglycan.degradation	KETO	T0	T2	0.016	0.157	↑	0.94	0.32
Other.transporters	KETO	T0	T3	0.016	0.157	↓	-0.12	0.04
Others	KETO	T0	T3	0.016	0.157	↓	-0.09	0.03
Ubiquinone.and.other.terpenoid.quinone.biosynthesis	KETO	T0	T2	0.016	0.157	↑	0.70	0.24
Retinol.metabolism	KETO	T0	T2	0.017	0.160	↑	0.88	0.31
Cell.cycle...Caulobacter	KETO	T0	T3	0.019	0.179	↓	-0.04	0.01
Prenyltransferases	KETO	T0	T3	0.020	0.181	↑	0.16	0.06
Glycan.biosynthesis.and.metabolism	KETO	T0	T3	0.021	0.188	↓	-0.36	0.13
D.Glutamine.and.D.glutamate.metabolism	KETO	T0	T3	0.022	0.190	↓	-0.20	0.08

Ethylbenzene.degradation	KETO	T0	T2	0.021	0.190	↓	-0.75	0.27
Polyketide.sugar.unit.biosynthesis	KETO	T0	T2	0.022	0.190	↓	-0.13	0.05
Drug.metabolism...cytochrome.P450	KETO	T0	T2	0.022	0.191	↑	0.84	0.31
Metabolism.of.xenobiotics.by.cytochrome.P450	KETO	T0	T2	0.022	0.191	↑	0.84	0.31
alpha.Linolenic.acid.metabolism	KETO	T0	T2	0.023	0.195	↑	0.78	0.31
Folate.biosynthesis	KETO	T0	T2	0.023	0.195	↑	0.16	0.06
Other.glycan.degradation	KETO	T0	T2	0.024	0.197	↑	0.57	0.21
Amino.acid.metabolism	KETO	T0	T3	0.025	0.206	↑	0.28	0.11
Bacterial.motility.proteins	KETO	T0	T2	0.027	0.206	↑	0.49	0.20
D.Glutamine.and.D.glutamate.metabolism	KETO	T0	T2	0.026	0.206	↓	-0.19	0.08
Lipid.biosynthesis.proteins	KETO	T0	T3	0.026	0.206	↓	-0.08	0.03
Lysosome	KETO	T0	T3	0.026	0.206	↑	0.79	0.30
Other.transporters	KETO	T0	T2	0.026	0.206	↓	-0.11	0.04
Retinol.metabolism	KETO	T0	T3	0.028	0.211	↑	0.79	0.31
Geraniol.degradation	KETO	T0	T3	0.028	0.212	↓	-0.66	0.26
Nucleotide.metabolism	KETO	T0	T3	0.029	0.213	↑	0.13	0.05
Others	KETO	T0	T2	0.029	0.213	↓	-0.08	0.03
Phenylalanine.metabolism	KETO	T2	T3	0.028	0.213	↓	-0.15	0.06
General.function.prediction.only	KETO	T0	T2	0.029	0.214	↓	-0.04	0.02
Benzoate.degradation	KETO	T0	T2	0.031	0.215	↓	-0.44	0.17
Cell.cycle...Caulobacter	KETO	T0	T2	0.032	0.215	↓	-0.03	0.01
Flagellar.assembly	KETO	T0	T3	0.031	0.215	↑	0.87	0.35
Glycosphingolipid.biosynthesis...ganglio.series	KETO	T0	T2	0.030	0.215	↑	0.77	0.31
Ribosome.Biogenesis	KETO	T0	T2	0.032	0.215	↓	-0.05	0.02
Sporulation	KETO	T0	T2	0.032	0.215	↓	-0.26	0.10
Toluene.degradation	KETO	T0	T2	0.032	0.215	↑	0.58	0.23
Two.component.system	KETO	T0	T3	0.032	0.215	↑	0.13	0.05
Xylene.degradation	KETO	T0	T2	0.032	0.215	↓	-0.75	0.30

Statistical significance was evaluated by running a Generalized Linear Mixed-effects Model with MaAsLin2. Effect-size is represented by the MaAsLin2 model coefficients (coeff.) and respective standard errors (std err.). Only predicted function abundance changes at p≤ 0.05 and q≤ 0.25 are considered statistically significant. q: p adjusted for Benjamini-Hochberg (BH) correction test with cut-off at q≤ 0.25. KETO= 6 patients who followed a very low-calorie ketogenic diet (VLCKD). Samples were analyzed at baseline (T0), after two months (T2) and after three months (T3) of the of nutritional intervention. ↓= significantly reduced in the group 1; ↑= significantly increased in the group 1.
